# Supplementary material for: Quantifying the tissue-specific regulatory information within enhancer DNA sequences
Source: NAR Genom Bioinform. 2021 Oct 27;3(4):lqab095. doi: 10.1093/nargab/lqab095 (PMC8557370; doi:10.1093/nargab/lqab095)
Supplement: lqab095_Supplemental_File [file lqab095_supplemental_file.pdf]

1 Supplementary Figures and Tables

1.1 SLR classifier

We tested a second type of logistic regression, called SLR in the following, which is based on motif scores. For the SLR classifier we use transcription factor binding motifs from the JASPAR 2018 database [1], which contains $m = 119$ motifs for mus musculus. Given the DNA sequence of a candidate regulatory element, we compute the maximum motif score for each of the m motifs. All scores are standardized before the coefficients θ are estimated.

We first tested the performance of the SLR classifier on active enhancers from the clustered collection (Supplementary Fig. 2). The SLR classifier with a median area under precision recall curve (PR-AUC) of 0.65 performs much worse than the KLR and SVM classifiers, with a median PR-AUC of 0.75 and 0.73 respectively. One reason for the poor performance of the SLR classifier could be that the set of motifs is incomplete. However, it may also be the case that motifs were estimated with inaccurate methods [2].

1.2 Discriminating between active and primed enhancers

It has been observed from genome-wide analyses of histone marks that enhancers can be in several different states. For instance, primed enhancers are lacking histone mark H3K27ac and are thought to be activated later during development [3, 4, 5, 6, 7]. We tested whether the KLR classifier is able to discriminate between primed and active enhancers within a tissue. Our results show similar classification performances for primed enhancers (Supplementary Fig. 7) as for active enhancers across all tissues. We extracted code words predictive for active enhancers (Supplementary Tables 18 to 25) and found that many are similar to known transcription factor motifs.

1.3 Location of the cell type-specific regulatory code

To compare classification results across different types of regulatory elements (i.e. active CpG-low/CpG-high promoters and active enhancers), it is important to have an equal number of positive and negative samples of each type. We therefore constructed a *balanced data collection* (Table 1) consisting of three different data sets. We constructed the data collection so that each set has 800 positive and 800 negative samples for each type of regulatory element. The data collection contains only three data sets, because most CpG-high promoters are differential between brain and non-brain tissues. We use the CpG ratio with a threshold of 0.4 to separate CpG-high from CpG-low promoters.

data set	positive	negative
1	forebrain midbrain hindbrain	kidney lung
2	forebrain midbrain hindbrain	limb liver
3	forebrain liver	heart kidney limb lung

Supplementary Table 1: **Definition of balanced data collection.**

Several different types of regulatory elements are known, each having a different role in the regulatory program. For instance, genes with high phyletic age tend to be expressed in many cell types and typically have promoters with high CpG dinucleotide content (CpG-high) [8]. Their regulatory program is likely to be different from that of other promoters since the expression of target genes can be silenced by methylation of the CpG dinucleotides within the promoter [9]. On the other hand, genes that evolved more recently tend to be tissue-specific and their promoters

show a low CpG dinucleotide content (CpG-low) [8]. Additionally, much evidence exists to support the idea that cell type-specific expression is mainly regulated by enhancers [10, 6].

To verify these observations, we trained and tested the KLR classifier separately on CpG-high and CpG-low promoters as well as active enhancers from the balanced data collection. Comparing the performances of the three sets of classifiers allows us to speculate about how the regulatory code is distributed over the elements. Indeed, we observe that the classification performances vary strongly across the three types of regulatory elements (Fig. 1). The performance is highest for enhancers and CpG-high promoters. This result suggests that the regulatory code is mainly embedded in enhancers, which then drive the activity of CpG-low promoters. CpG-high promoters are known to be a special case, since they can be silenced through CpG methylation [11]. Our results suggest that the regulatory code for CpG-high promoters is, at least to some extent, present in the promoter DNA sequences and not outsourced to enhancers. However, we also know that the distribution of the CpG ratio of active promoters varies greatly between tissues (Supplementary Figs. 7 and 8) [12], with brain tissues having the highest CpG ratios.

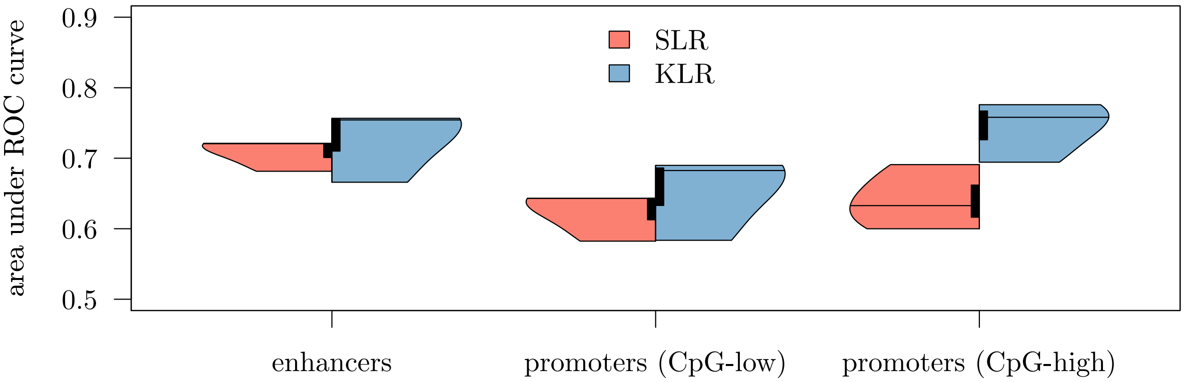

Supplementary Figure 1: **Classifier performances stratified by type of regulatory elements.** The figure shows the distribution of area under ROC-curves using 10-fold cross-validation for the SLR and KLR classifiers

1.4 Importance of combinatorial binding of transcription factors

It is well established that in eukaryotes, interactions between transcription factors are essential for the regulation of gene expression, either by direct protein-protein interactions between factors or by interactions through the basal transcription machinery [13, 14, 15, 16, 17]. The combinatorics of transcription factor binding within regulatory elements have been studied extensively for promoters [18, 19] and enhancers [20]. We are interested in determining to what extent combinatorial binding is important for predicting cell type-specific activity of regulatory elements. To investigate this question, we extended the SLR and KLR classifiers to include interaction terms.

Both the KLR and SLR classifiers can model co-occurrences by adding interaction terms to the logistic regression, i.e. we define

$$x \odot \theta = \theta_0 + x_{i1}\theta_1 + \cdots + x_{im}\theta_m + x_{i1}x_{i2}\theta_{m+1} + x_{i1}x_{i3}\theta_{m+1} + \cdots + x_{im-1}x_{im}\theta_{(m+1)m/2} \,,$$

where $\theta \in \mathbb{R}^{(m+1)m/2+1}$. For the KLR classifier, $m = 156,570$ so that $(m+1)m/2 = 12,257,160,735$, which is computationally too expensive to estimate. Instead, we first select the 1000 most important

code words and consider co-occurrences only between those, resulting in a parameter vector θ of dimension $500500 + 1$. This procedure is equivalent to assuming *strong hierarchy*, which is not only computationally necessary, but often argued to be sensible [21].

We compared the classification performance of both classifiers with and without interaction terms on the leaves data collection. Our results show that there is no significant difference between the two types of classifiers (Fig. 2), although classifiers that model co-occurrences show mainly non-zero coefficients for interaction terms. For the SLR classifier we observe a very marginal increase in classification performance on enhancers when interactions are considered, but the opposite is the case for the KLR classifier. This result might suggest that combinatorial binding is not important for cell type-specific activity of regulatory elements. However, it may also be the case that transcription factors generally co-bind in close proximity so that this effect is already captured by the SLR and KLR classifiers without interaction terms.

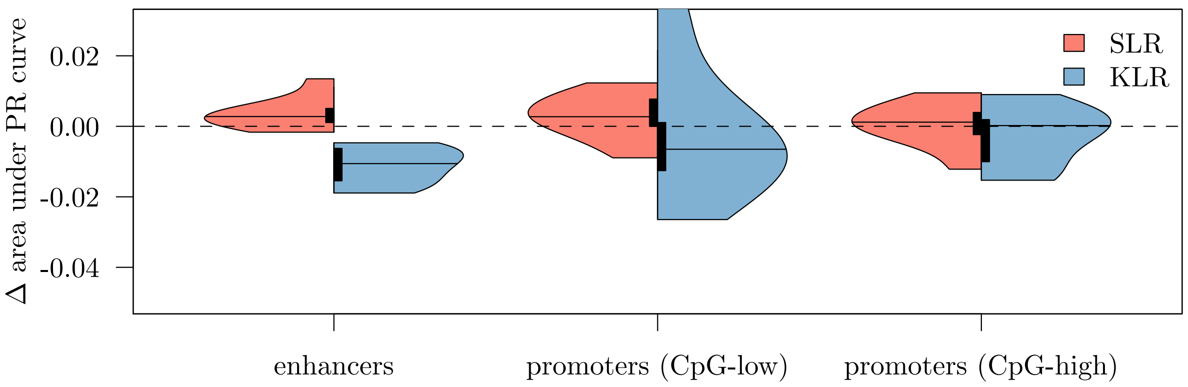

Supplementary Figure 2: **Importance of combinatorial binding.** Difference in area under precision recall (PR) curve between classifiers with and without interaction terms

1.5 Mitochondrial Footprint Control

We use the mitochondrial genome to account for possible sequence specific biases of transposase Tn5. The mitochondrial genome is everywhere accessible to proteins and because of its abundance shows a high coverage of ATAC-seq reads. To compute the mitochondrial control footprint, we first identify code word occurrences on the mitochondrial genome. The ATAC-seq signal in a window of 500 bps around code word occurrences is normalized by its mean and averaged across all n code word occurrences. In addition, we compute the variance σ_i^2 of the signal at every window position $i \in \{1, 2, \ldots, 500\}$ to account for the relatively short length of the mitochondrial genome. The variance of the mean $\hat{\sigma}_i^2 = \sigma_i^2/n$ is reported as a confidence interval of two standard deviations around the mean.

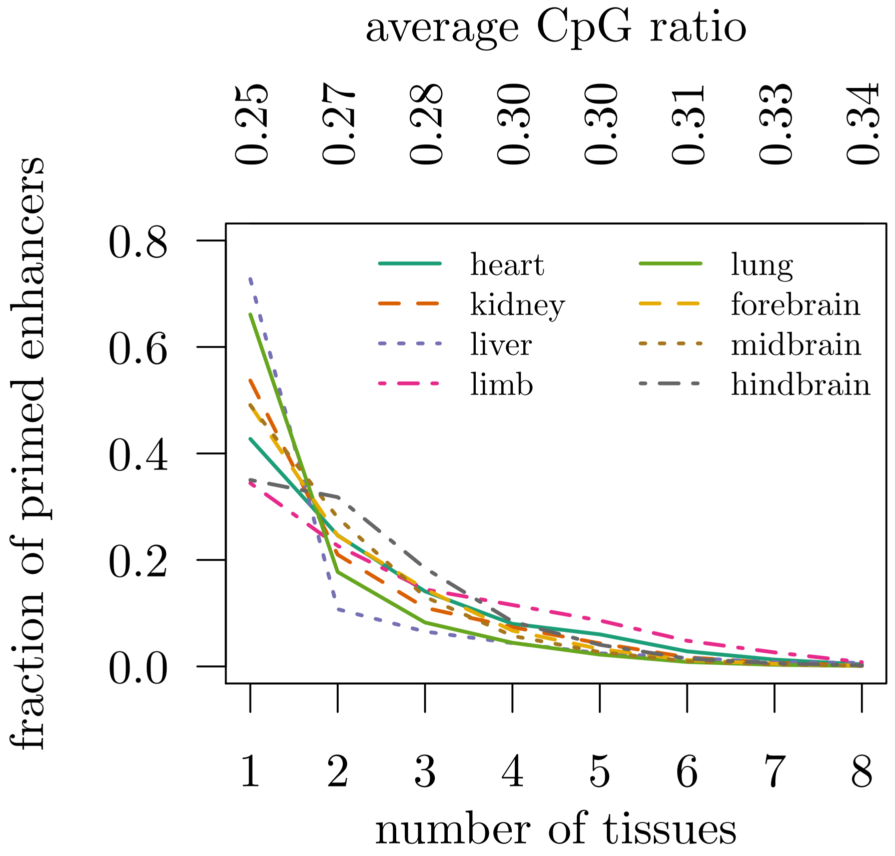

Supplementary Figure 3: Tissue specificity of primed enhancers

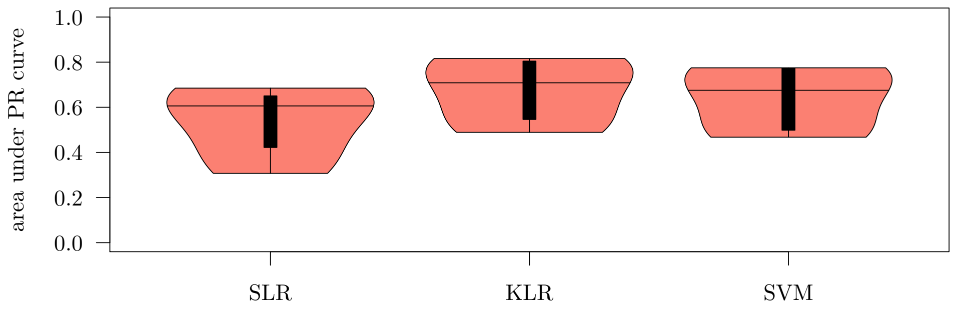

Supplementary Figure 4: Classifier performance evaluated on clustered data collection of active enhancers

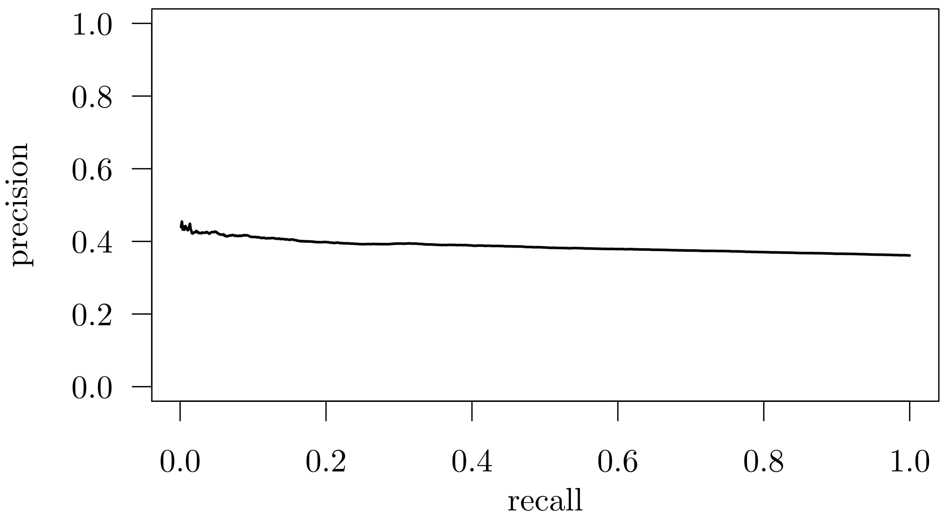

Supplementary Figure 5: KLR classifier performance on control data set

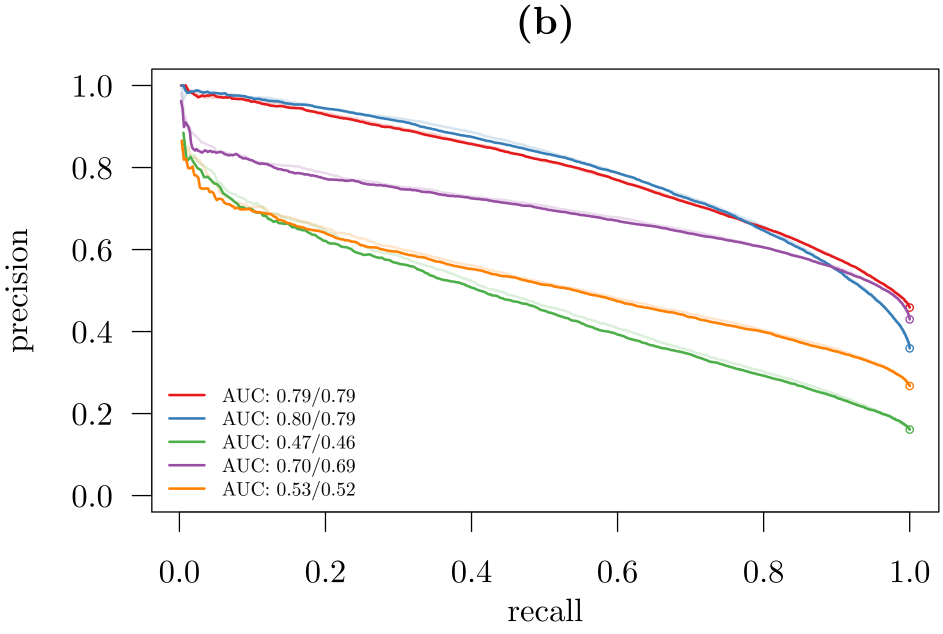

Supplementary Figure 6: KLR classifier performance on clustered data collection using cross-validation with test sets sorted by chromosome position. Curves with light colors show chross-validation with randomized genomic regions, solid lines correspond to cross-validation using sorted genomic regions

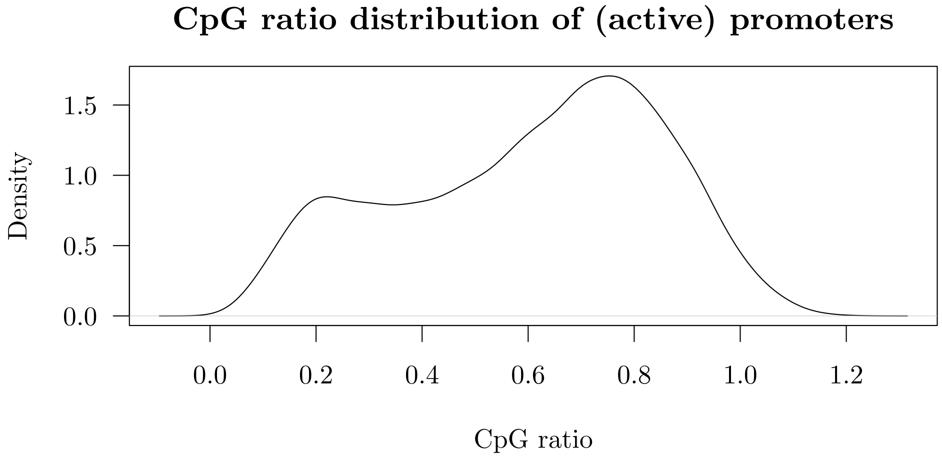

Supplementary Figure 7: CpG ratio distribution of promoters, active in at least one tissue

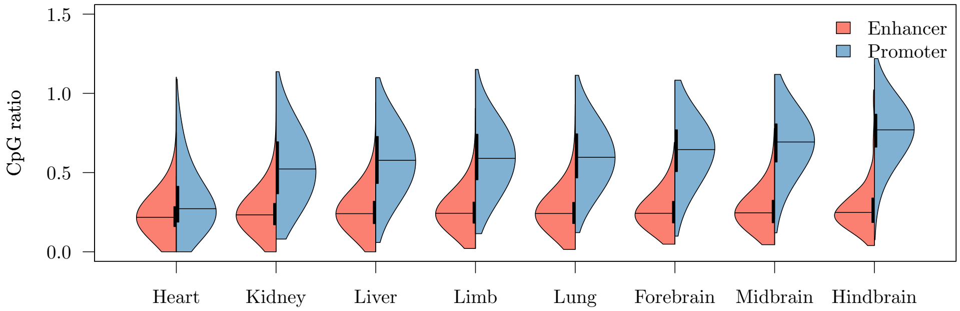

Supplementary Figure 8: CpG ratio of active enhancers and promoters stratified by tissue

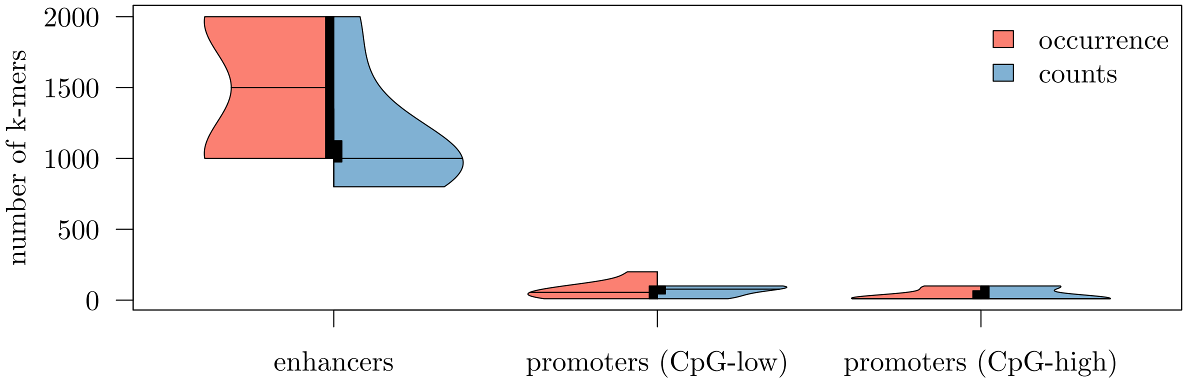

Supplementary Figure 9: Number of features used by the optimal KLR classifier when using code word occurrences or counts

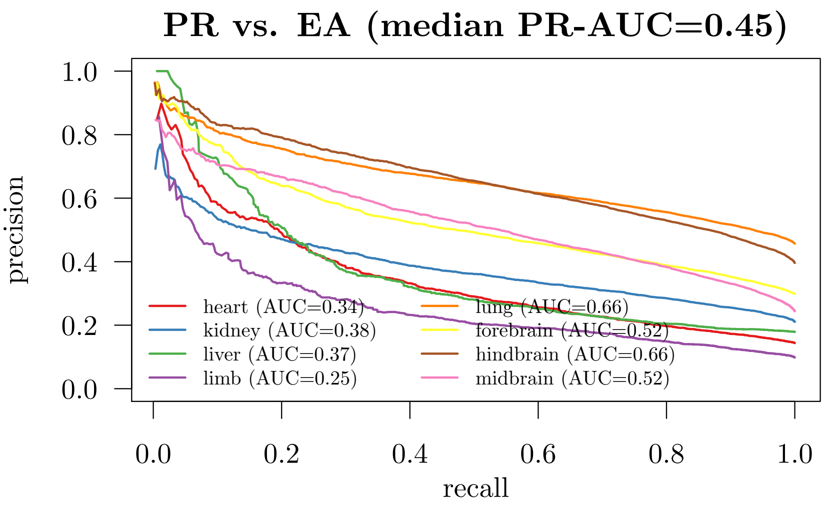

Supplementary Figure 10: KLR classification performance on primed (PR) versus active (EA) enhancers within tissues

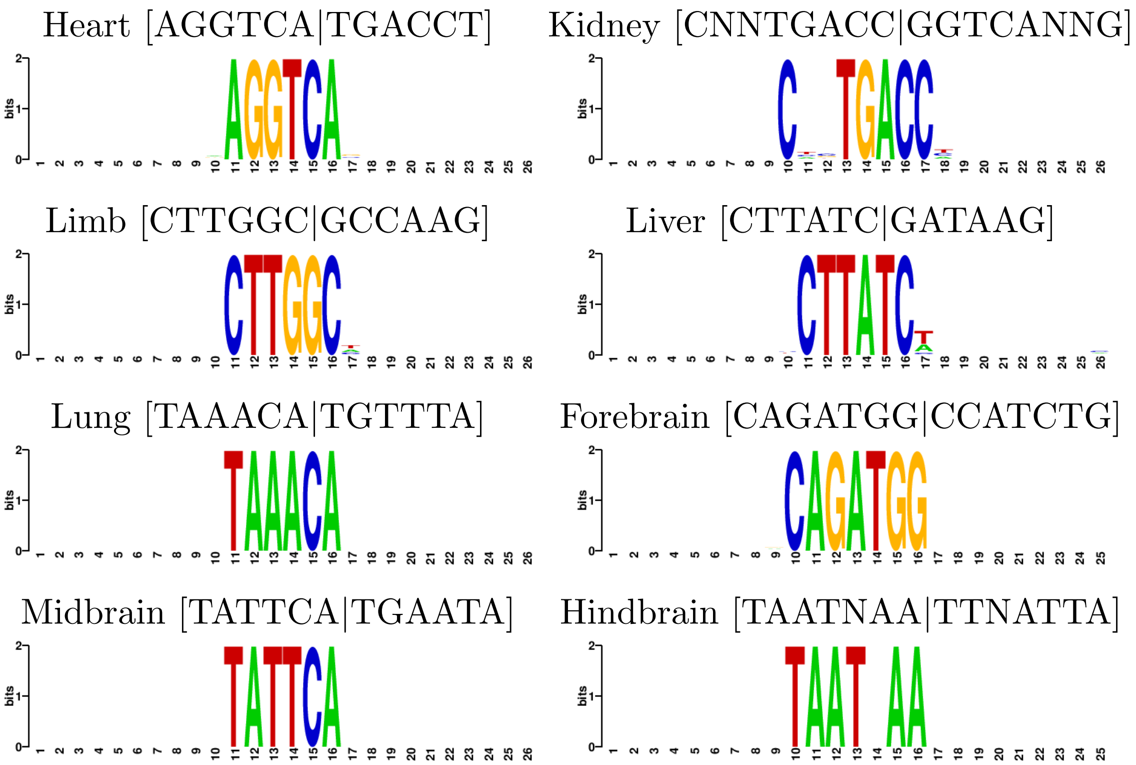

Supplementary Figure 11: Sequence logos of code word neighborhoods in active enhancers.

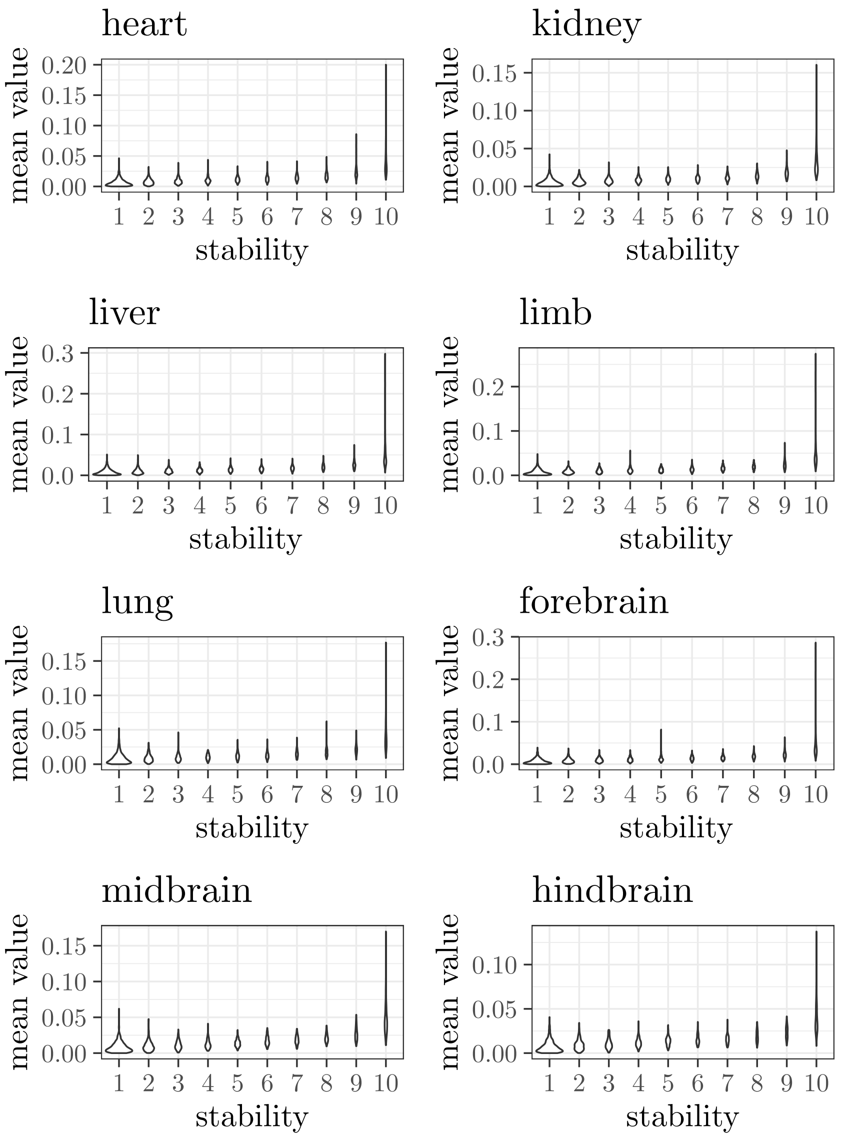

Supplementary Figure 12: Stability of code words across cross-validation (CV) runs on the leaves data collection. The x-axis shows in how many out of the 10 CV iterations a code word had a non-zero coefficient. The y-axis shows for each detected code-word the mean absolute value of the coefficient across CV iterations. For computing the mean, we excluded iterations where the code word has a coefficient of zero. We observe that code words with high mean absolute value are stable across all CV iterations.

Coefficient	k-mer
1.220076e-01	CNGCNCNG|CNGNGCNG
1.133865e-01	CNGCNGNC|GNCNGCNG
1.104353e-01	CCCNGCC|GGCNGGG
1.092400e-01	GCNCNGC|GCNGNGC
9.447861e-02	CNGNCAGC|GCTGNCNG
9.439475e-02	CCGNNC|GNNCGG
8.421516e-02	CNGCNGC|GCNGCNG
8.076685e-02	CNCCNCCC|GGGNGGNG
8.025976e-02	CGNNNGC|GCNNNCG
7.957235e-02	CGCNG|CNGCG
7.945013e-02	CAGNGCNG|CNGCNCTG
7.579287e-02	CNCNCNGC|GCNGNGNG
7.021016e-02	CCTNCCNG|CNGGNAGG
6.449539e-02	CGNGC|GCNCG
5.339787e-02	CNGGCTNC|GNAGCCNG
4.705916e-02	CAGCNCNG|CNGNGCTG
4.445257e-02	GCNGCNC|GNGCNGC
4.402429e-02	CNNGCNGC|GCNGCNNG
4.110281e-02	CGNNCNG|CNGNNCG
4.090604e-02	CTGGCNC|GNGCCAG

Supplementary Table 2: Heart active enhancer versus random k-mers

Coefficient	k-mer
4.092643e-02	ATATNNAA|TTNNATAT
4.082406e-02	ATATANA|TNTATAT
3.410699e-02	AANATAT|ATATNTT
1.913669e-02	TATNANAA|TTNTNATA
1.152353e-02	ATATNTA|TANATAT
8.781651e-03	ANATATA|TATATNT
8.651638e-03	ATATTNT|ANAATAT
8.373656e-03	ATATAA|TTATAT
6.013567e-03	ANNATATA|TATATNNT
3.185595e-03	ATATA|TATAT
2.389406e-03	ATATANNT|ANNTATAT
5.172689e-04	AANANTAT|ATANTNTT
1.364710e-04	ATATNNAT|ATNNATAT
4.621480e-05	CNTATA|TATANG
4.850692e-07	AATNNTAT|ATANNATT

Supplementary Table 3: Kidney active enhancer versus random k-mers

Coefficient	k-mer
7.844098e-02	ATTTNNAT|ATNNAAAT
6.227846e-02	AAAATC|GATTTT
3.799466e-02	AATATNNA|TNNATATT
1.514132e-02	ATANAAT|ATTNTAT
1.126232e-02	AANNATAT|ATATNNTT
9.860305e-03	AATNNTAT|ATANNATT
4.661901e-03	AANATAT|ATATNTT
3.743103e-03	ATTNNATA|TATNNAAT
3.002073e-03	ATATNAA|TTNATAT
6.330809e-04	ATATTNNT|ANNAATAT

Supplementary Table 4: Liver active enhancer versus random k-mers

Coefficient	k-mer
2.287901e-02	ATANNTAA|TTANNTAT
1.607827e-02	ATATNTA|TANATAT
1.129433e-02	ATATAA|TTATAT
9.325971e-03	AATATNNA|TNNATATT
5.390299e-03	ATANANAA|TTNTNTAT
2.456531e-03	AANATNTA|TANATNTT
1.127811e-03	ANATATNA|TNATATNT
1.026810e-03	ATATA|TATAT
9.111476e-04	ANNATATA|TATATNNT
6.708790e-04	TATNANAA|TTNTNATA
1.751466e-07	ANATATA|TATATNT

Supplementary Table 5: Limb active enhancer versus random k-mers

Coefficient	k-mer
5.287803e-02	ATATANNT|ANNTATAT
3.219219e-02	ATNNAATA|TATTNNAT
2.952235e-02	AATNNTAT|ATANNATT
2.121272e-02	ATATAA|TTATAT
1.992908e-02	AANATAT|ATATNTT
1.321708e-02	ATATNNAA|TTNNATAT
1.032477e-02	ATATNNAT|ATNNATAT
7.143787e-03	TATNANAA|TTNTNATA
3.685859e-03	ATATTNT|ANAATAT
7.770563e-04	ATNTATNA|TNATANAT
6.815450e-04	ATAANAT|ATNTTAT
5.183059e-05	CNTATA|TATANG

Supplementary Table 6: Lung active enhancer versus random k-mers

Coefficient	k-mer
7.705880e-02	CNTATA|TATANG
3.225029e-02	CATATA|TATATG
1.789592e-02	ATATANA|TNTATAT
2.945417e-03	GAGTTNNA|TNNAACTC
5.286091e-07	ATATANNT|ANNTATAT

Supplementary Table 7: Forebrain active enhancer versus random k-mers

Coefficient	k-mer
4.967507e-02	AAAGTT|AACTTT
3.015293e-02	CNTATA|TATANG
2.849713e-02	ATATANA|TNTATAT
2.261469e-02	AAACTT|AAGTTT
1.191651e-02	GAGTTNNA|TNNAACTC
8.910725e-03	AACTTNT|ANAAGTT
8.839285e-03	CATATA|TATATG

Supplementary Table 8: Midbrain active enhancer versus random k-mers

Coefficient	k-mer
5.341277e-02	CNTATA|TATANG
3.697316e-02	ANATATA|TATATNT
3.657011e-02	ATATANA|TNTATAT
2.917223e-02	AANATAT|ATATNTT
3.764890e-03	AAAGTT|AACTTT
1.539902e-04	AANANTAT|ATANTNTT
2.499315e-05	AAAANTT|AANTTTT

Supplementary Table 9: Hindbrain active enhancer versus random k-mers

Coefficient	k-mer
1.882018e-01	CTTATC|GATAAG
1.638524e-01	AGGTCA|TGACCT
9.075613e-02	AAAATAG|CTATTTT
8.853232e-02	CAAGGNCA|TGNCCTTG
7.665507e-02	AANATAG|CTATNTT
5.752587e-02	TATNTNTA|TANANATA
5.717503e-02	ANATAGC|GCTATNT
5.676097e-02	TATTTNTA|TANAAATA
4.573678e-02	CNTGTCA|TGACANG
4.471814e-02	ANGTCAC|GTGACNT
4.285185e-02	CNGATA|TATCNG
3.984399e-02	ANNAATAG|CTATTNNT
3.829973e-02	GNTGACA|TGTCANC
3.185298e-02	CNTATC|GATANG
3.184707e-02	GTCANCA|TGNTGAC
2.997837e-02	AGATANNG|CNNTATCT
2.782536e-02	CNNGTGAC|GTCACNNG
2.629641e-02	ATTNNTAG|CTANNAAT
2.530363e-02	ANGGTCA|TGACCNT
2.286028e-02	AGTNCTG|CAGNACT

Supplementary Table 10: Heart active enhancer k-mers

Coefficient	k-mer
1.020531e-01	CNNTGACC|GGTCANNG
9.948961e-02	ATTAAC|GTTAAT
9.763349e-02	AGGTCA|TGACCT
7.191567e-02	ANNGGTCA|TGACCNNT
6.905743e-02	GGGTCA|TGACCC
6.754502e-02	CTCCCAC|GTGGGAG
6.525444e-02	AAGTTNNA|TNNAACTT
6.500284e-02	GATTTA|TAAATC
6.387318e-02	CATNAANC|GNTTNATG
3.620010e-02	GTNAATNA|TNATTNAC
3.502837e-02	CTGACC|GGTCAG
3.428181e-02	CCATNAA|TTNATGG
3.282915e-02	AGTTNANG|CNTNAACT
3.228190e-02	CNNGGTCA|TGACCNNG
3.212338e-02	CAGGTNA|TNACCTG
3.144703e-02	GGTTAA|TTAACC
2.510691e-02	CCNCCCNC|GNGGGNGG
2.445600e-02	CNTGACC|GGTCANG
2.394907e-02	GTTAANNA|TNNTTAAC
2.385617e-02	AAGTTNA|TNAACTT

Supplementary Table 11: Kidney active enhancer k-mers

Coefficient	k-mer
4.418726e-01	CTTATC|GATAAG
3.842613e-01	AGATAA|TTATCT
2.415544e-01	TGATAA|TTATCA
2.166643e-01	AGATANG|CNTATCT
1.408881e-01	GGTGGNNC|GNNCCACC
1.024764e-01	CTGATA|TATCAG
8.786420e-02	ANAGATA|TATCTNT
7.655353e-02	AGTTCNA|TNGAACT
7.446016e-02	AGATANNG|CNNTATCT
6.929535e-02	AGTTNNAG|CTNNAACT
5.718874e-02	GAGTTCNA|TNGAACTC
5.654989e-02	GAGATA|TATCTC
5.442684e-02	CNACCAC|GTGGTNG
4.427998e-02	AGATANNA|TNNTATCT
4.313449e-02	CNTTATC|GATAANG
4.220056e-02	AGATANC|GNTATCT
4.088724e-02	CACACCC|GGGTGTG
4.069697e-02	ANNAGATA|TATCTNNT
3.259381e-02	ANGNCTAC|GTAGNCNT
2.730205e-02	GTAGANNA|TNNTCTAC

Supplementary Table 12: Liver active enhancer k-mers

Coefficient	k-mer
8.756674e-02	CTTGGC|GCCAAG
8.739274e-02	ANTTTCCA|TGGAAANT
6.196472e-02	GCCAANNC|GNNTTGGC
4.770059e-02	CNTGGNCC|GGNCCANG
4.538072e-02	ACNTNTGG|CCANANGT
3.698461e-02	TGGAAAA|TTTTCCA
3.471553e-02	ACATCTG|CAGATGT
3.331488e-02	TGGAAANA|TNTTTCCA
3.251135e-02	TTCCANA|TNTGGAA
2.754918e-02	CATNTGG|CCANATG
2.203030e-02	AAACTT|AAGTTT
2.069077e-02	CCAGANNC|GNNTCTGG
1.509852e-02	ANACCNCA|TGNGGTNT
1.388453e-02	CNATAAA|TTTATNG
1.365222e-02	ANNCCAGA|TCTGGNNT
9.218143e-03	CCNATAA|TTATNGG
8.995484e-03	CANACCC|GGGTNTG
7.366801e-03	CNNGCCAA|TTGGCNNG
3.353828e-03	GGCCCA|TGGGCC
3.290024e-03	CANCTGG|CCAGNTG

Supplementary Table 13: Limb active enhancer k-mers

Coefficient	k-mer
1.406340e-01	TAAACA|TGTTTA
8.770747e-02	GTAAAC|GTTTAC
8.654490e-02	TGTTTANA|TNTAAACA
7.529245e-02	CAAACA|TGTTTG
5.069352e-02	ANATTCC|GGAATNT
5.057327e-02	ACTTCNNG|CNNGAAGT
4.514979e-02	AAGTNCT|AGNACTT
3.844192e-02	AAACTT|AAGTTT
3.057111e-02	ANNTAAAC|GTTTANNT
2.795234e-02	AGNAAACA|TGTTTNCT
2.753212e-02	ATAAACA|TGTTTAT
2.531748e-02	TTTGNTNA|TNANCAAA
2.358802e-02	ACTTNTG|CANAAGT
2.319156e-02	TTNCANAA|TTNTGNAA
2.287478e-02	ANNGTTTA|TAAACNNT
2.190120e-02	GTTTGNNC|GNNCAAAC
2.184071e-02	AAGTNNCT|AGNNACTT
2.159117e-02	ANTNTTTG|CAAANANT
2.139472e-02	ACATTCC|GGAATGT
2.057346e-02	ACTTCNT|ANGAAGT

Supplementary Table 14: Lung active enhancer k-mers

Coefficient	k-mer
3.284869e-01	CAGATGG|CCATCTG
1.747920e-01	CAATTA|TAATTG
1.558061e-01	ATTNGCA|TGCNAAT
1.048507e-01	CNAATTA|TAATTNG
8.073584e-02	TAATTA|TAATTA
7.885150e-02	AATTAG|CTAATT
6.729461e-02	ATGNNAAT|ATTNNCAT
5.550338e-02	ACANATNG|CNATNTGT
4.861347e-02	ATGCNAA|TTNGCAT
4.517273e-02	ATGAAT|ATTCAT
4.471312e-02	ANNATATG|CATATNNT
3.985233e-02	TGCCAA|TTGGCA
3.959699e-02	CATTTNNA|TNNAAATG
3.744615e-02	ANNTGGCA|TGCCANNT
3.647300e-02	AATTANNC|GNNTAATT
3.586182e-02	CATATG|CATATG
3.507460e-02	ATTTNNAT|ATNNAAAT
3.461155e-02	AATTANC|GNTAATT
3.446622e-02	CTNATTA|TAATNAG
2.912524e-02	AATNNGCA|TGCNNATT

Supplementary Table 15: Forebrain active enhancer k-mers

Coefficient	k-mer
1.194402e-01	TATTCA|TGAATA
1.092740e-01	AATAAT|ATTATT
8.937580e-02	ATGNNAAT|ATTNNCAT
8.665814e-02	GATNAAA|TTTNATC
8.247386e-02	ATTTNNAT|ATNNAAAT
6.004712e-02	ATTTNAA|TTNAAAT
4.700935e-02	ATCAAA|TTTGAT
3.942919e-02	ATTATNC|GNATAAT
3.730211e-02	ATTATNNA|TNNATAAT
2.954801e-02	AATNNAAT|ATTNNATT
2.938446e-02	ATNAAANG|CNTTTNAT
2.914561e-02	AAAATC|GATTTT
2.613495e-02	CTNATTA|TAATNAG
2.508567e-02	ATNAATA|TATTNAT
2.357498e-02	AATCNNTT|AANNGATT
2.307671e-02	ATNAAAG|CTTTNAT
2.175419e-02	ATNNATTA|TAATNNAT
1.905055e-02	TAATGA|TCATTA
1.504929e-02	ATNAANAG|CTNTTNAT
1.206887e-02	ATTANNNC|GNNNTAAT

Supplementary Table 16: Midbrain active enhancer k-mers

Coefficient	k-mer
6.310558e-02	TAATNAA|TTNATTA
4.995234e-02	ATNNATCA|TGATNNAT
4.090126e-02	CNNTGGCA|TGCCANNG
3.064466e-02	CGNNNAGC|GCTNNNCG
3.004314e-02	AGCNNNCG|CGNNNGCT
2.948802e-02	AATTGNNT|ANNCAATT
2.872896e-02	CNCGCA|TGCGNG
2.674954e-02	CTAATNA|TNATTAG
2.322091e-02	CANCGC|GCGNTG
2.315027e-02	ATNCATC|GATGNAT
1.798161e-02	GCATCNNC|GNNGATGC
1.349962e-02	CNCTGCNG|CNGCAGNG
1.281059e-02	ATGGCA|TGCCAT
1.233782e-02	CGNTGC|GCANCG
1.200482e-02	GCNGCTNC|GNAGCNGC
1.046248e-02	ANGATGC|GCATCNT
1.003060e-02	CTGCNGNC|GNCNGCAG
9.450373e-03	CATNNATC|GATNNATG
8.122928e-03	GCAGNGC|GCNCTGC
7.055018e-03	AGCGA|TCGCT

Supplementary Table 17: Hindbrain active enhancer k-mers

Coefficient	k-mer
7.108817e-02	AAATAG|CTATTT
6.639535e-02	GAAAANNA|TNNTTTTC
4.611863e-02	ATTTNNAG|CTNNAAAT
1.874686e-02	TAANAANA|TNTTNTTA
1.087803e-02	ANAAATA|TATTTNT
7.252695e-03	AAAATA|TATTTT
5.869543e-03	TAAAANNA|TNNTTTTA
4.795092e-03	AAANAGNA|TNCTNTTT
4.711524e-03	ANNTTTAG|CTAAANNT
3.249960e-03	TATTTNNA|TNNAAATA
2.100815e-03	TTTCNAA|TTNGAAA

Supplementary Table 18: Heart active/primed enhancer k-mers

Coefficient	k-mer
2.595118e-02	AGGAANT|ANTTCCT
2.510978e-02	CANGAAA|TTTCNTG
2.186890e-02	CANNAAGC|GCTTNNTG
1.809457e-02	ANATTCC|GGAATNT
1.584056e-02	GNAAACA|TGTTTNC
1.066339e-02	AANTTCC|GGAANTT
7.775262e-03	ATTTCC|GGAAAT
7.707218e-03	AGGNAGGA|TCCTNCCT
3.535195e-03	CNGGAAA|TTTCCNG
2.838993e-03	GNAGGAA|TTCCTNC
1.873522e-03	CAGGAAG|CTTCCTG
1.480342e-03	AAANNAAC|GTTNNTTT
6.165058e-04	AAACAA|TTGTTT
4.955818e-04	GCNGGAA|TTCCNGC

Supplementary Table 19: Kidney active/primed enhancer k-mers

Coefficient	k-mer
2.726096e-02	CNCAGNAA|TTNCTGNG
2.466940e-02	ANACAAG|CTTGTNT
2.289408e-02	GCCTNAC|GTNAGGC
1.448976e-02	ACANGCC|GGCNTGT
1.275854e-02	AGGCCNNT|ANNGGCCT
7.445750e-03	GGNCNACA|TGTNGNCC
4.608107e-03	AGCTNAG|CTNAGCT
1.362636e-03	ANAGNAAC|GTTNCTNT

Supplementary Table 20: Liver active/primed enhancer k-mers

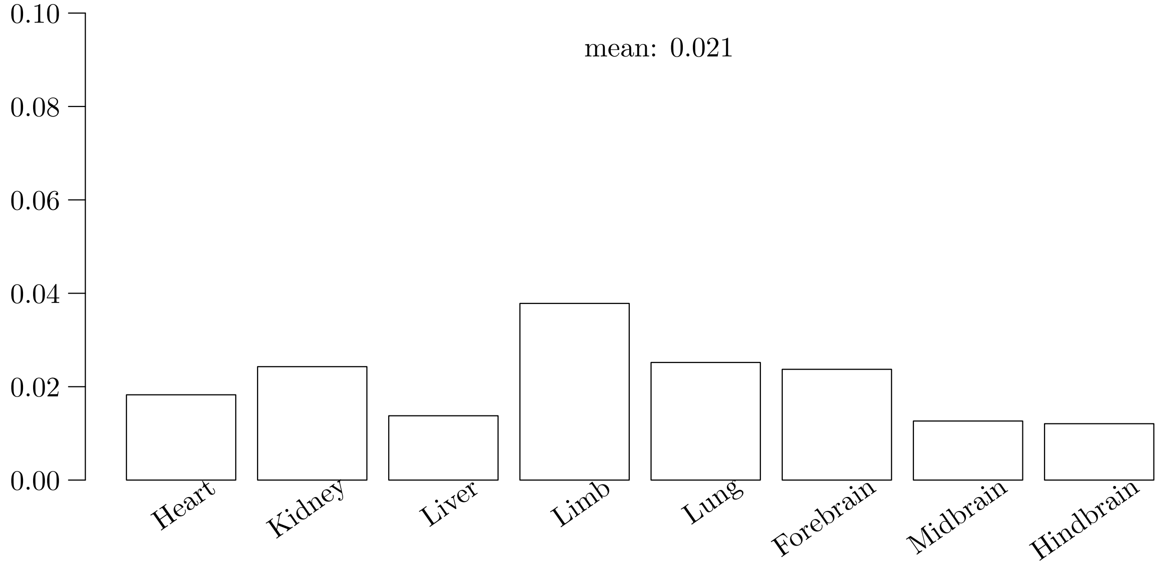

Supplementary Figure 13: **Code word counts versus occurrences.** The figure shows the difference in area under precision recall (PR) curve when code word occurrences are used as opposed to code word counts. PR curves are evaluated for the KLR classifier using 10-fold cross-validation

Coefficient	k-mer
4.946880e-02	AGNAAANC|GNTTTNCT
4.142856e-02	AAANAGNC|GNCTNTTT
3.357433e-02	TGGAAA|TTTCCA
3.274859e-02	ANNTTTCC|GGAAANNT
2.919853e-02	ANNTAAAA|TTTTANNT
2.623187e-02	ANTTTCC|GGAAANT
1.958511e-02	GAANGNAA|TTNCNTTC
1.947151e-02	AGTTNNTG|CANNAACT
1.596530e-02	GNTTTNCA|TGNAAANC
1.355715e-02	TGAANNAA|TTNNTTCA
1.086766e-02	TTNAGNAA|TTNCTNAA
1.032791e-02	AGANTTNC|GNAANTCT
9.988572e-03	AAANCAA|TTGNTTT
9.082434e-03	TGNNGAAA|TTTCNNCA
7.522810e-03	AANCTCT|AGAGNTT
5.293159e-03	AAANNTCT|AGANNTTT
3.609649e-03	AGNAAANA|TNTTTNCT
3.455022e-03	ANAGGAA|TTCCTNT
2.498589e-03	ATTTNCNT|ANGNAAAT
6.208859e-04	GGAAANA|TNTTTCC

Supplementary Table 21: Limb active/primed enhancer k-mers

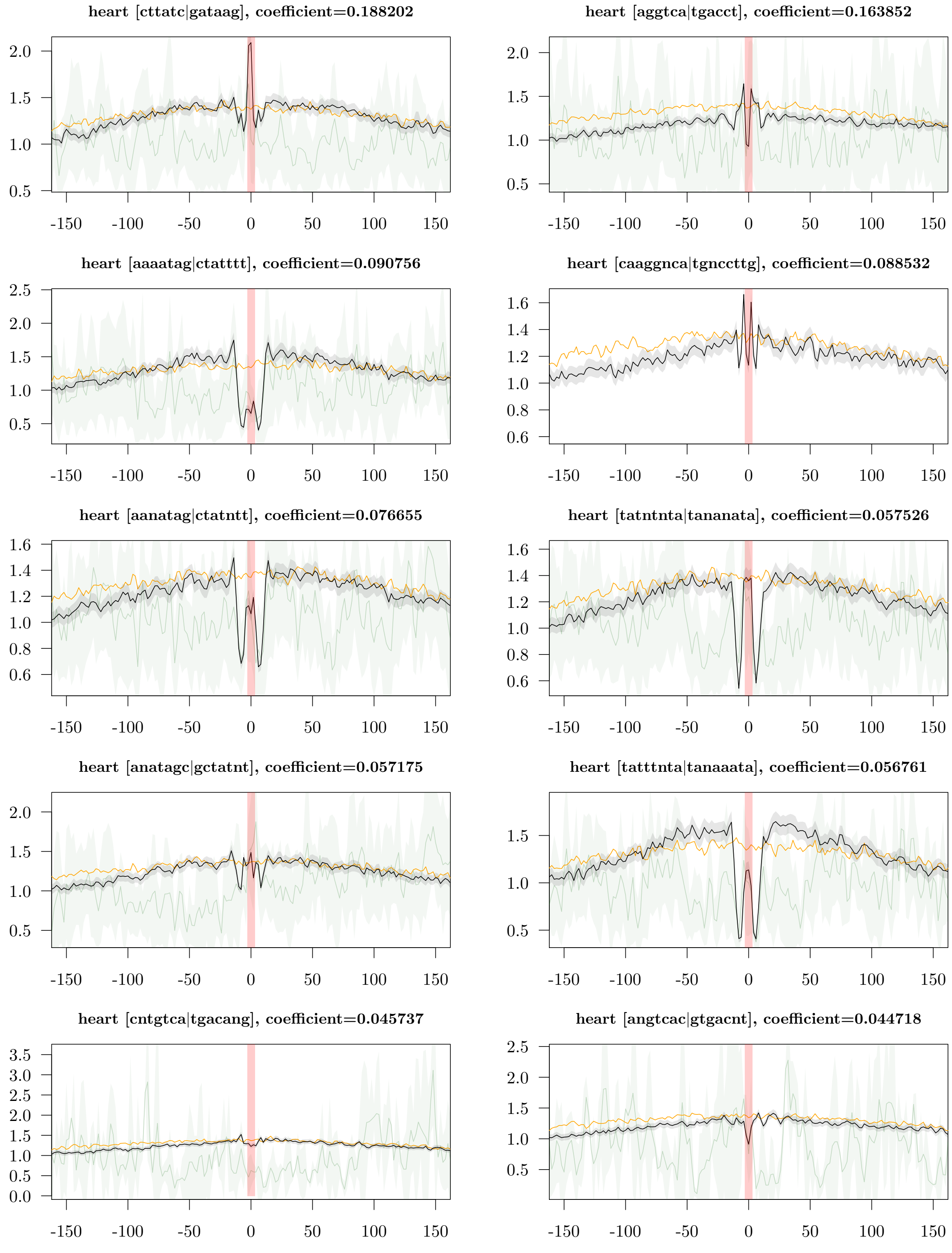

Supplementary Figure 14: ATAC-seq footprints around 10 most important code words of active enhancers (black lines) in heart. The control (gray lines) shows the average ATAC-seq signal of the same regions, but aligned at the center of the enhancer elements.

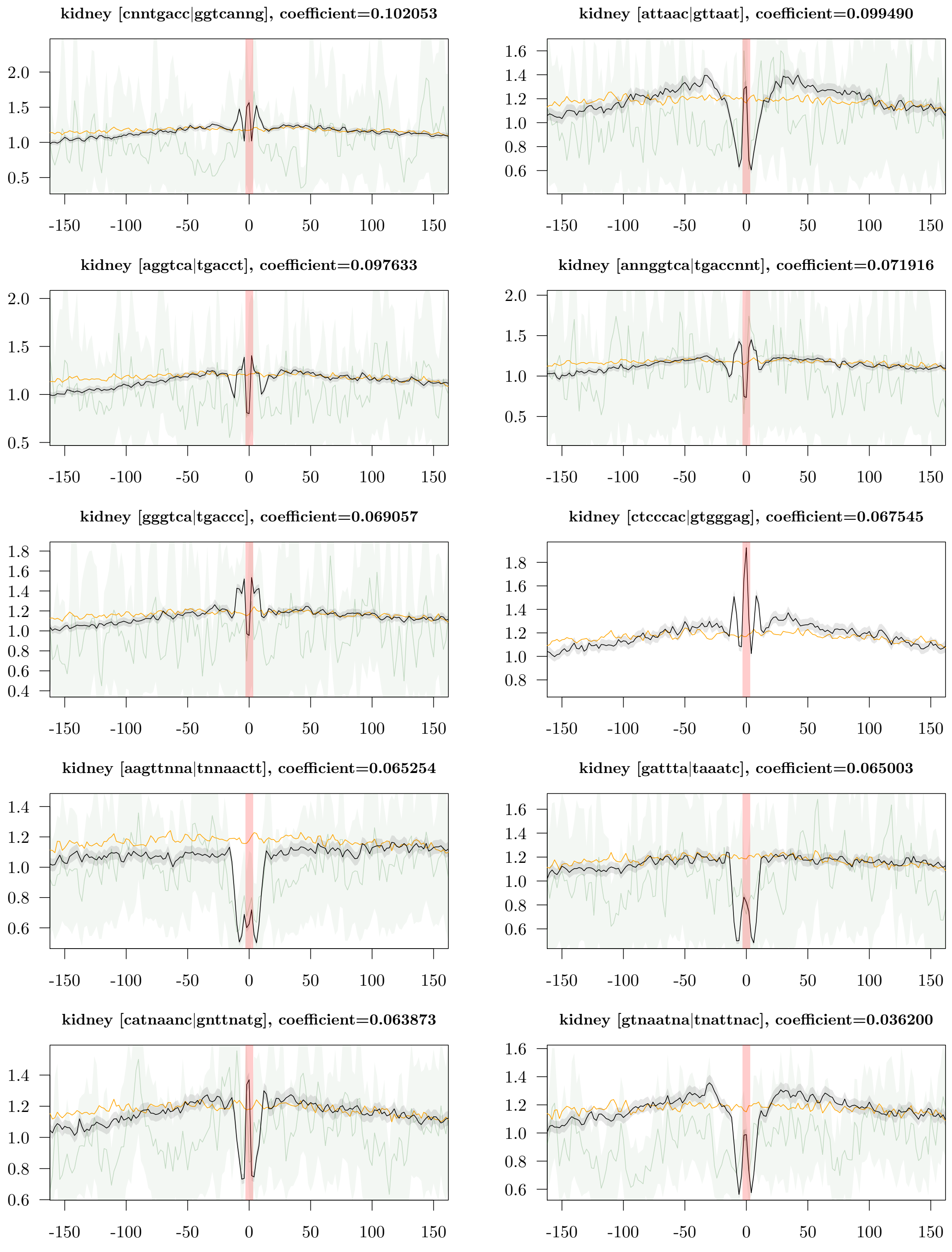

Supplementary Figure 15: ATAC-seq footprints around 10 most important code words of active enhancers (black lines) in kidney. The control (gray lines) shows the average ATAC-seq signal of the same regions, but aligned at the center of the enhancer elements.

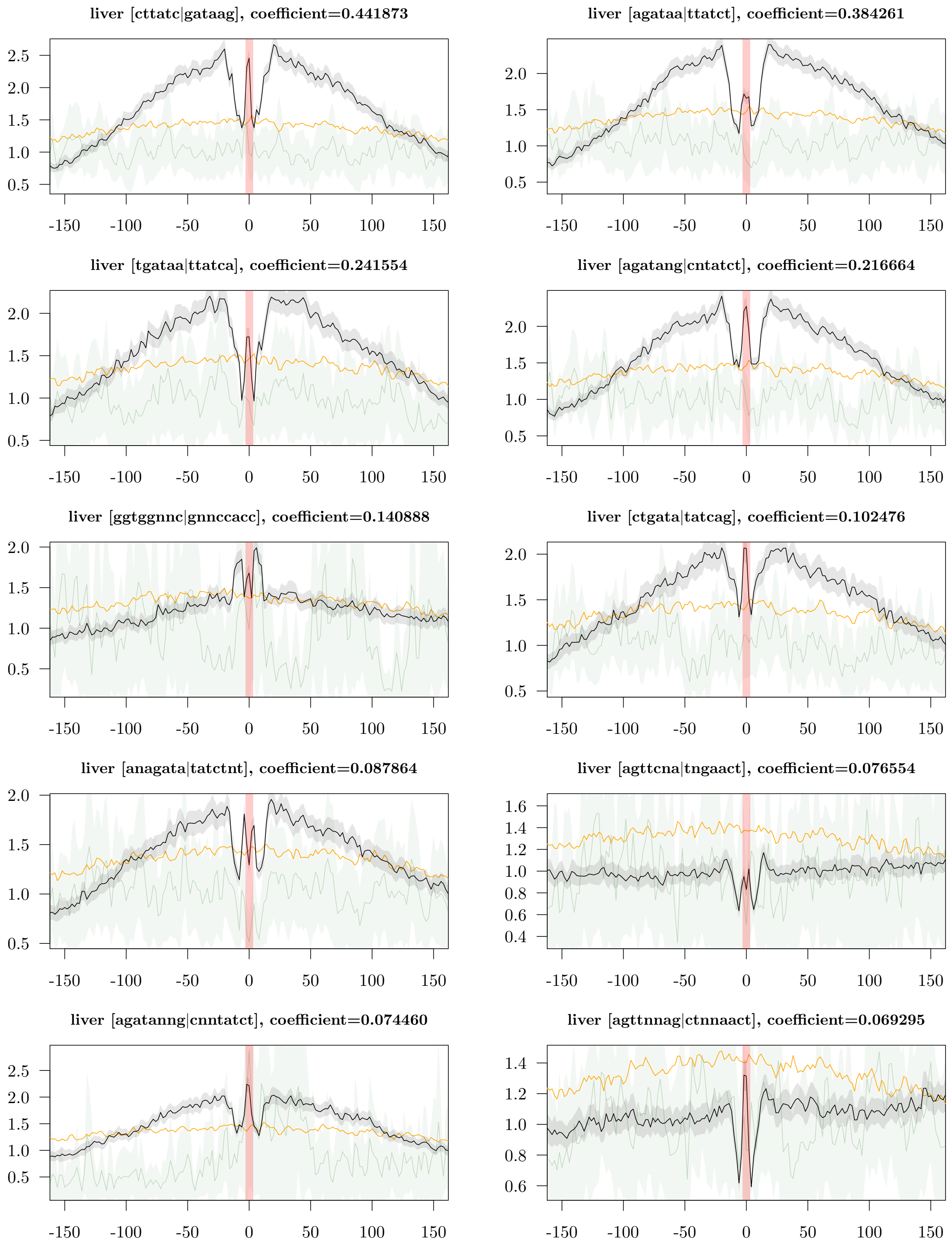

Supplementary Figure 16: ATAC-seq footprints around 10 most important code words of active enhancers (black lines) in liver. The control (gray lines) shows the average ATAC-seq signal of the same regions, but aligned at the center of the enhancer elements.

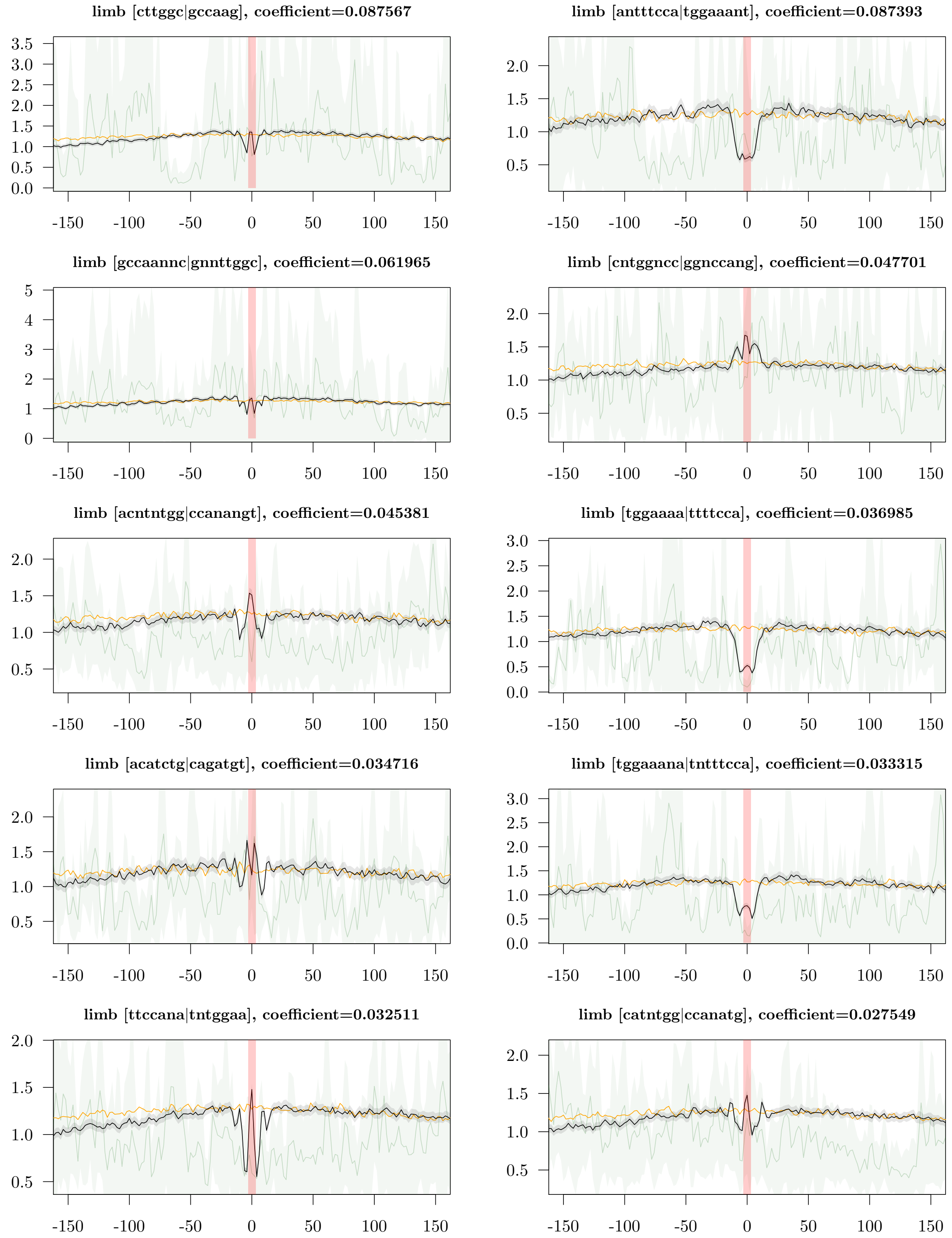

Supplementary Figure 17: ATAC-seq footprints around 10 most important code words of active enhancers (black lines) in limb. The control (gray lines) shows the average ATAC-seq signal of the same regions, but aligned at the center of the enhancer elements.

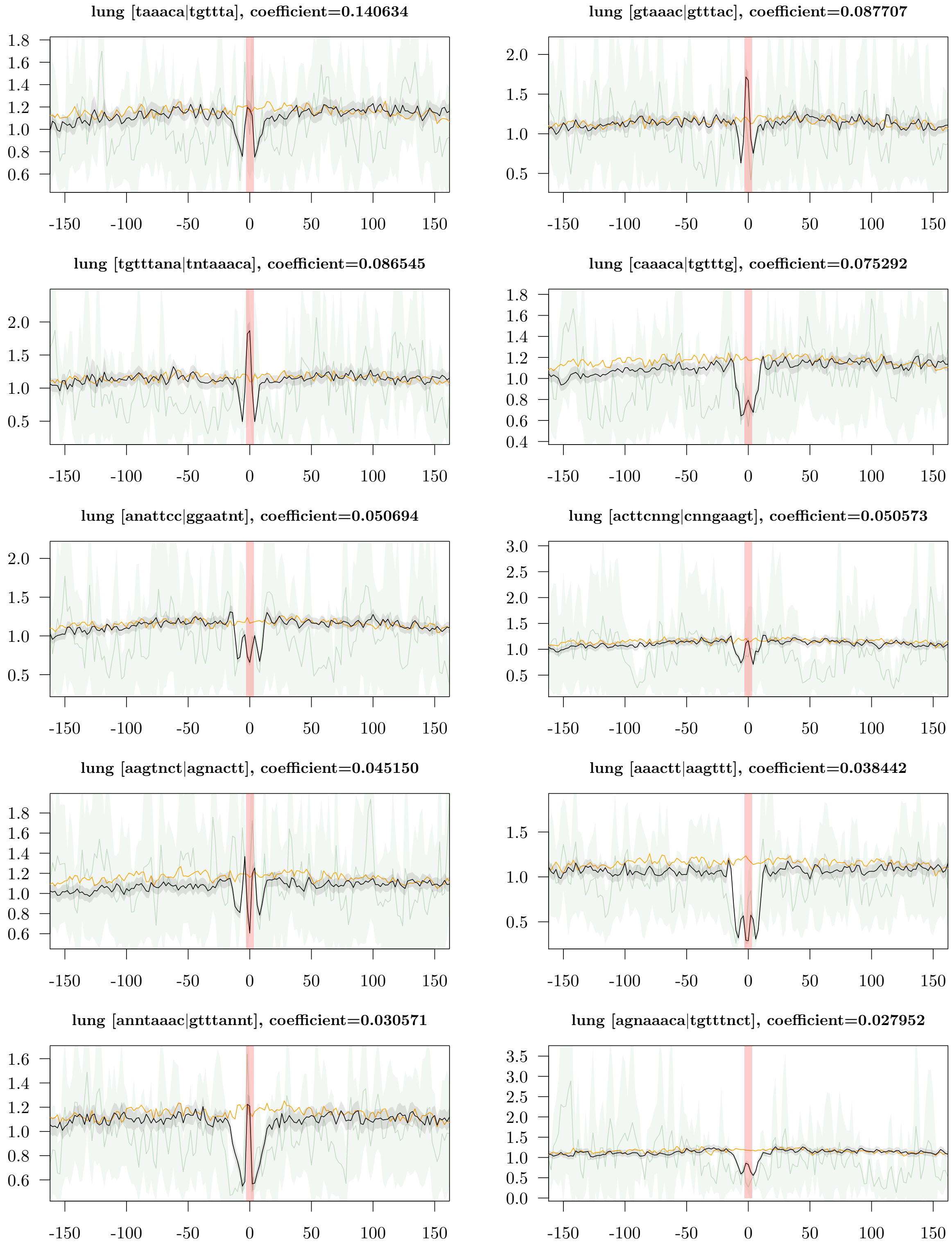

Supplementary Figure 18: ATAC-seq footprints around 10 most important code words of active enhancers (black lines) in lung. The control (gray lines) shows the average ATAC-seq signal of the same regions, but aligned at the center of the enhancer elements.

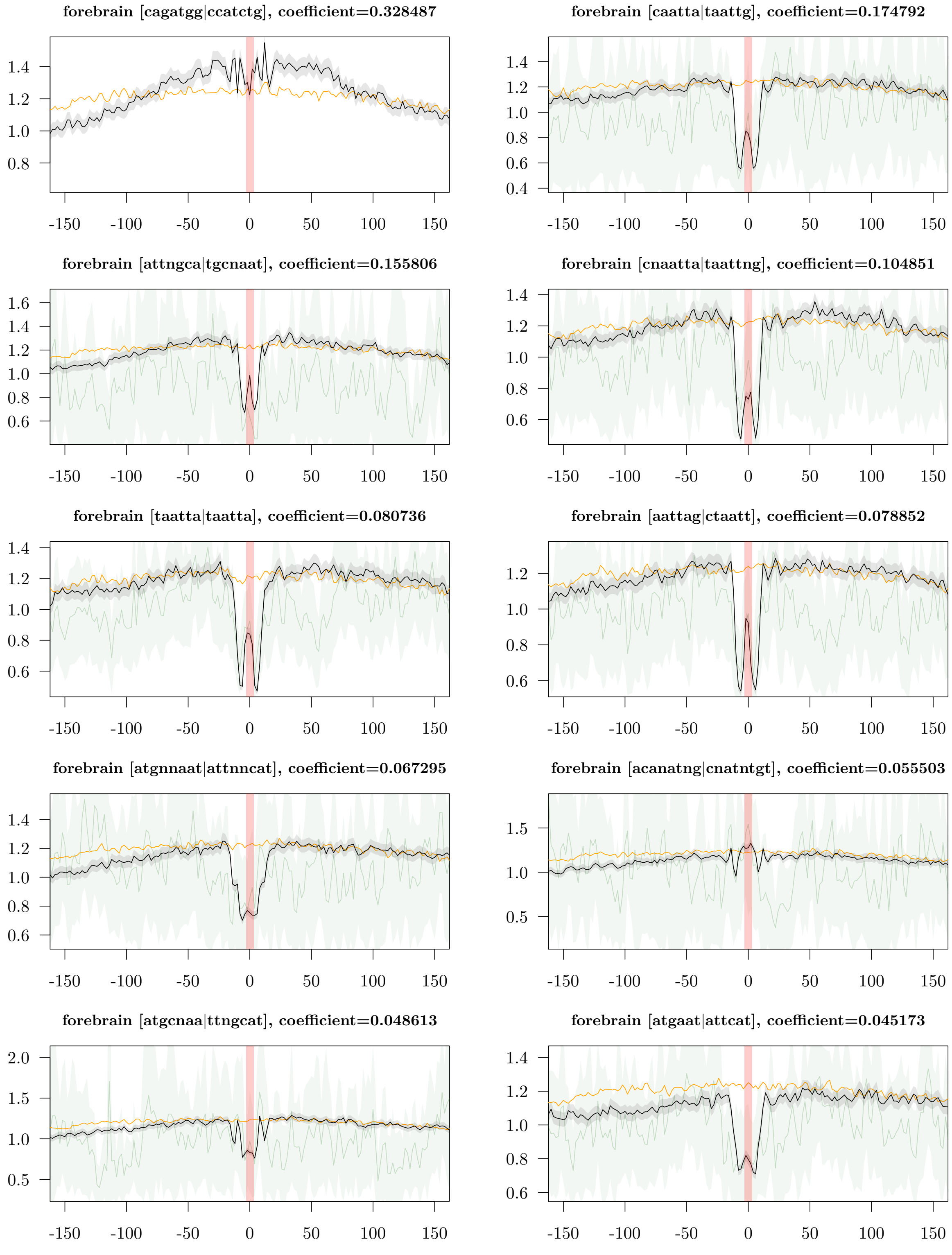

Supplementary Figure 19: ATAC-seq footprints around 10 most important code words of active enhancers (black lines) in forebrain. The control (gray lines) shows the average ATAC-seq signal of the same regions, but aligned at the center of the enhancer elements.

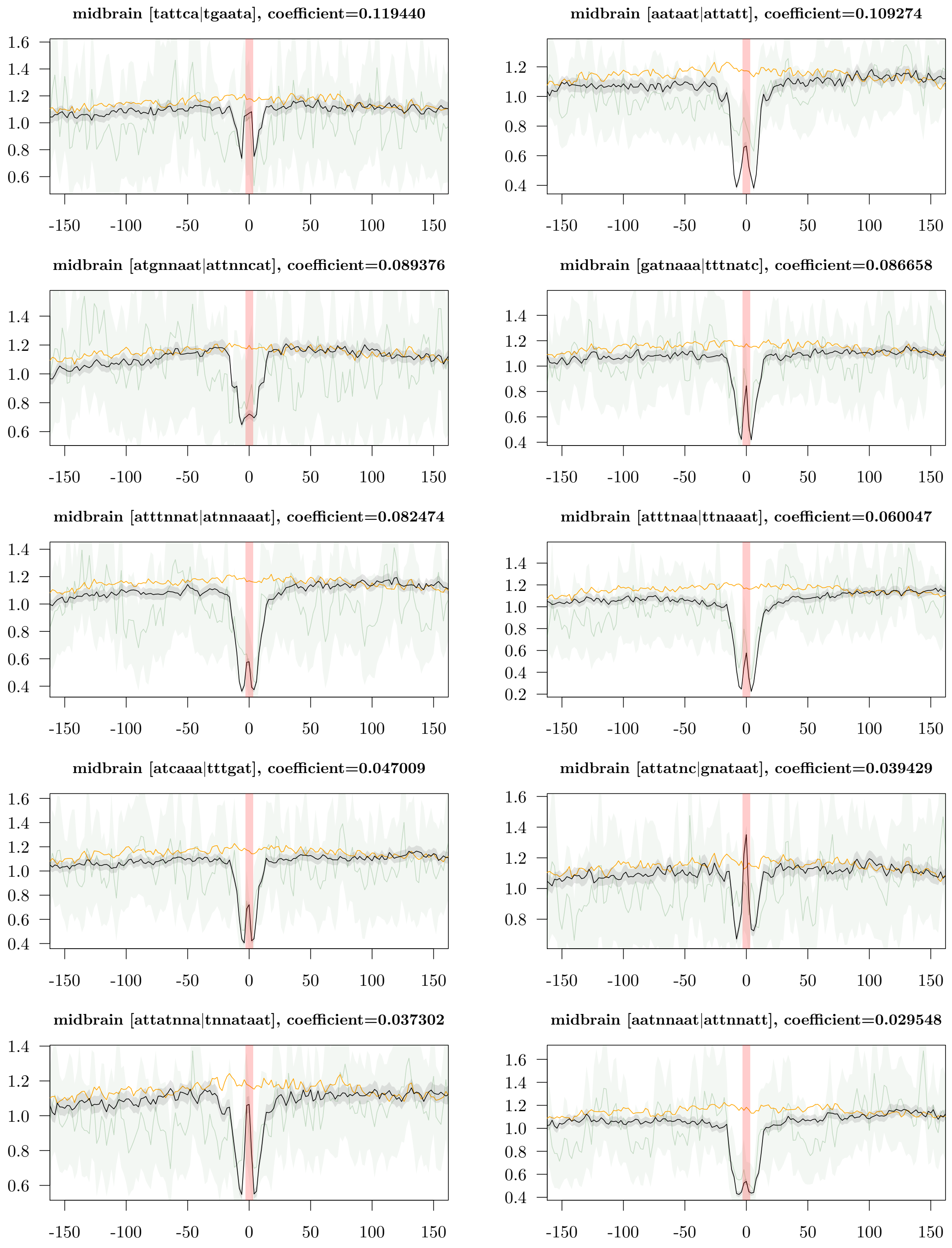

Supplementary Figure 20: ATAC-seq footprints around 10 most important code words of active enhancers (black lines) in midbrain. The control (gray lines) shows the average ATAC-seq signal of the same regions, but aligned at the center of the enhancer elements.

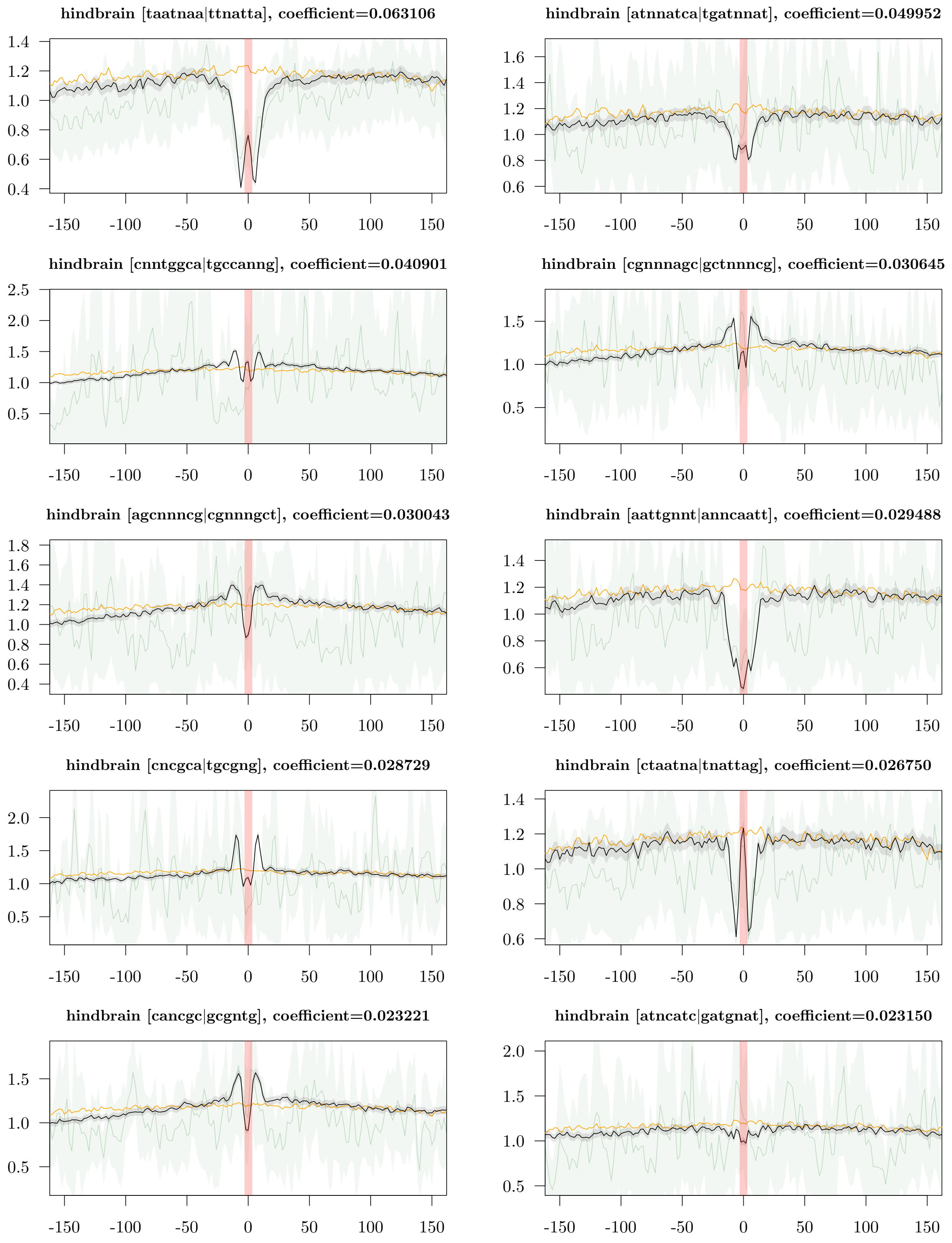

Supplementary Figure 21: ATAC-seq footprints around 10 most important code words of active enhancers (black lines) in hindbrain. The control (gray lines) shows the average ATAC-seq signal of the same regions, but aligned at the center of the enhancer elements.

Coefficient	k-mer
3.954595e-02	GNAAACA|TGTTTNC
3.450363e-02	AGNAAAA|TTTTNCT
3.376044e-02	AGGAANT|ANTTCCT
2.923563e-02	AGNAAAT|ATTTNCT
1.775037e-02	ANAGGAA|TTCCTNT
1.648291e-02	AGANNAAA|TTTNNTCT
1.585723e-02	AAACANT|ANTGTTT
1.585384e-02	ATTTCC|GGAAAT
1.367495e-02	AAANAGA|TCTNTTT
1.222750e-02	CAGGAAG|CTTCCTG
1.202030e-02	AANNAAAG|CTTTNNTT
1.124490e-02	AAACANNC|GNNTGTTT
1.064884e-02	AANAAANG|CNTTTNTT
6.117277e-03	ANTTTCC|GGAAANT
5.464630e-03	AGNAAAC|GTTTNCT
5.254366e-03	TTCNAAA|TTTNGAA
3.708424e-03	TGTTTNA|TNAAACA
3.179645e-03	AAAGNAA|TTNCTTT
2.036569e-03	AAGAAA|TTTCTT
1.867751e-03	TNTAAANA|TNTTTANA

Supplementary Table 22: Lung active/primed enhancer k-mers

Coefficient	k-mer
1.590400e-01	CAGATGG|CCATCTG
1.970634e-02	ANAAAGC|GCTTTNT
1.873085e-02	ACANANGG|CCNTNTGT
1.862789e-02	CANANAGC|GCTNTNTG
1.855113e-02	AANAAAGG|CCTTTNTT
1.467011e-02	ACAAANG|CNTTTGT
1.132120e-02	ACAAAG|CTTTGT
8.978300e-03	CNGCAGC|GCTGCNG
7.756535e-03	ANGCAGC|GCTGCNT
7.199301e-03	AGNCAGC|GCTGNCT
7.149984e-03	AGAGCT|AGCTCT
7.121278e-03	ACAAAGG|CCTTTGT
5.402761e-03	CANATGG|CCATNTG
4.022826e-03	GANNGAAA|TTTCNNTC
2.913987e-03	AANANAGA|TCTNTNTT
2.079053e-03	AGCTGNNA|TNNCAGCT
1.919643e-03	GNGAGANA|TNTCTCNC
1.884114e-03	GCTGNCA|TGNCAGC
1.654904e-03	AANGAGA|TCTCNTT
1.433402e-03	ANANAGCC|GGCTNTNT

Supplementary Table 23: Forebrain active/primed enhancer k-mers

Coefficient	k-mer
2.916380e-02	GAAANGC|GCNTTTC
1.987357e-02	CNGCAGC|GCTGCNG
1.884978e-02	ATTNGCA|TGCNAAT
1.586346e-02	GCAGCC|GGCTGC
1.317559e-02	ANAAANGC|GCNTTTNT
1.033002e-02	GTTGCNA|TNGCAAC
7.934415e-03	CNGCNGC|GCNGCNG
6.761922e-03	AGCNGCNG|CNGCNGCT
4.859020e-03	AGCTGC|GCAGCT
3.454828e-03	ANNGGAAA|TTTCCNNT
3.396658e-03	CNGNCAGC|GCTGNCNG
2.929552e-03	AAANAGC|GCTNTTT
2.127981e-03	AAANGAG|CTCNTTT
1.505571e-03	GCNGANAA|TTNTCNGC
1.428004e-03	ATTTGC|GCAAAT

Supplementary Table 24: Midbrain active/primed enhancer k-mers

Coefficient	k-mer
8.847729e-02	ACAAAG|CTTTGT
6.154593e-02	ACAAANG|CNTTTGT
2.916447e-02	GNAAATNA|TNATTTNC
2.555737e-02	AAANNGCT|AGCNNTTT
2.532082e-02	ATTTNCA|TGNAAAT
2.463892e-02	ANAAANGC|GCNTTTNT
2.092278e-02	GCTGCA|TGCAGC
2.031603e-02	AGCNGCNG|CNGCNGCT
1.533200e-02	GAANAAA|TTTNTTC
1.471883e-02	CNGCAGC|GCTGCNG
1.417746e-02	GAAANGC|GCNTTTC
1.376909e-02	ATTTCNNT|ANNGAAAT
1.224286e-02	CNTTTNTC|GANAAANG
1.195203e-02	AAANAGC|GCTNTTT
5.577006e-03	AAGCNGC|GCNGCTT
4.821936e-03	ATTTCC|GGAAAT
4.380671e-03	AANGANGC|GCNTCNTT
4.255284e-03	ANGCAGC|GCTGCNT
3.652331e-03	AACAANG|CNTTGTT
3.599566e-03	GAAANAAA|TTTNTTTC

Supplementary Table 25: Hindbrain active/primed enhancer k-mers

Tissue	Code word	Motif matches
heart	CTTATC|GATAAG	GATA4 (3.57565e-05), GATA6 (3.57565e-05), GATA1 (8.75558e-05), TAL1 (0.000107266), GATA2 (0.000189313)
heart	AGGTCA|TGACCT	NR4A2 (2.97522e-05), NR1H3 (3.71901e-05), NR4A3 (3.71901e-05), RXRB (3.71901e-05), ERR1 (5.20657e-05)
heart	AAAATAG|CTATTTT	MEF2C (7.55259e-06), MEF2A (2.45025e-05), MEF2D (4.7644e-05), NR1D1 (0.00142363), HMGA1 (0.00191669)
heart	CAAGGNCA|TGNCCTTG	NR5A2 (3.77687e-06), STF1 (3.77687e-06), ERR1 (4.72109e-06), ERR2 (9.44216e-06), ERR3 (1.02333e-05)
heart	AANATAG|CTATNTT	MEF2C (6.18798e-05), MEF2A (0.000174815), MEF2D (0.000314118), FOXD1 (0.00484009), NR1D1 (0.00623547)
heart	TATNTNTA|TANANATA	MEF2D (0.000147604), MEF2A (0.000468521), MEF2C (0.000792277), FOXJ3 (0.00255984), FOXD1 (0.00413634)
heart	ANATAGC|GCTATNT	MEF2D (0.000853188), MEF2A (0.00212515), MEF2C (0.00285844), TYY1 (0.0105332), VSX2 (0.0105332)
heart	TATTTNTA|TANAAATA	MEF2D (2.06836e-05), MEF2A (7.70666e-05), MEF2C (0.00013809), FOXJ3 (0.00132059), SOX5 (0.00230172)
heart	CNTGTCA|TGACANG	TBX20 (0.000784641), PBX1 (0.000971622), PKNX1 (0.00137819), XBP1 (0.00145021), MEIS1 (0.00287528)
heart	ANGTCAC|GTGACNT	ERR3 (0.000210706), RXRB (0.000253346), NR4A2 (0.000294313), E4F1 (0.000538482), ATF1 (0.000548911)
kidney	CNNTGACC|GGTCANNG	RARA (3.86812e-05), NR1H2 (9.28323e-05), RXRA (0.000131841), VDR (0.000141369), NR2C2 (0.000145921)
kidney	ATTAAC|GTTAAT	HNF1B (5.50044e-05), HNF1A (6.87551e-05), HNF1B (6.87551e-05), LHX2 (0.00097381), PITX1 (0.00210951)
kidney	AGGTCA|TGACCT	NR4A2 (2.97522e-05), NR1H3 (3.71901e-05), NR4A3 (3.71901e-05), RXRB (3.71901e-05), ERR1 (5.20657e-05)
kidney	ANNGGTCA|TGACCNNT	PPARA (3.02812e-05), COT2 (4.46279e-05), NR4A3 (4.54214e-05), COT1 (4.69386e-05), NR4A2 (4.69386e-05)
kidney	GGGTCA|TGACCC	USF2 (0.000219899), USF1 (0.000271771), NR1D1 (0.00034045), NR1H4 (0.00121346), RARA (0.00133448)
kidney	CTCCCAC|GTGGGAG	WT1 (6.56076e-05), NR0B1 (0.000329828), ARNT2 (0.000435497), IKZF1 (0.000624289), EGR2 (0.000760802)
kidney	AAGTTNNA|TNNAACTT	ERR2 (0.000911985), HSF1 (0.00144414), RXRG (0.00256969), NR6A1 (0.00266097), RARA (0.00291858)
kidney	GATTTA|TAAATC	HXA9 (4.12536e-05), EVX1 (5.50044e-05), HXC6 (6.87551e-05), CDX4 (9.84533e-05), MEIS1 (0.000343607)
kidney	CATNAANC|GNTTNATG	HXB6 (4.29076e-05), HXB7 (0.000152561), PBX1 (0.000181163), MEIS1 (0.000203841), HXB8 (0.000244839)
kidney	GTNAATNA|TNATTNAC	HNF1B (8.43891e-05), HNF1B (0.000112517), PBX1 (0.000411167), HNF1A (0.000639533), LHX2 (0.000867229)
liver	CTTATC|GATAAG	GATA4 (3.57565e-05), GATA6 (3.57565e-05), GATA1 (8.75558e-05), TAL1 (0.000107266), GATA2 (0.000189313)
liver	AGATAA|TTATCT	EVI1 (7.56303e-05), GATA1 (0.000131114), GATA2 (0.000182447), GATA4 (0.000196335), GATA6 (0.000196335)
liver	TGATAA|TTATCA	GATA4 (0.00295586), GATA6 (0.00309589), GATA1 (0.00468026), GATA2 (0.00513277), GATA3 (0.00652879)
liver	AGATANG|CNTATCT	GATA1 (0.000157763), GATA4 (0.000163076), GATA6 (0.000163076), GATA2 (0.000203841), EVI1 (0.000315501)
liver	GGTGGNNC|GNNCCACC	GLI2 (0.00150364), KLF5 (0.0019411), GLI1 (0.00209918), GLI3 (0.00246025), KLF4 (0.00300501)
liver	CTGATA|TATCAG	GATA4 (0.00405912), SIX2 (0.00729455), LHX6 (0.00936169), GATA2 (0.00945329), GATA1 (0.010425)
liver	ANAGATA|TATCTNT	GATA3 (0.000559614), GATA4 (0.000722329), TAL1 (0.000970139), GATA2 (0.00112354), GATA5 (0.00126439)
liver	AGTTCNA|TNGAACT	ERR2 (0.000347046), RARA (0.000441674), RXRG (0.00106314), RXRG (0.00151274), RXRA (0.00159913)
liver	AGATANNG|CNNTATCT	GATA6 (0.000358093), GATA2 (0.0013449), GATA1 (0.00142115), GATA1 (0.00224636), GATA4 (0.00280092)
liver	AGTTNNAG|CTNNAACT	ERR2 (0.000315501), RARA (0.000410132), RXRG (0.000797463), RXRG (0.00141826), RXRA (0.0014926)
limb	CTTGGC|GCCAAG	NFIA (6.79496e-05), NFIC (0.000685512), TLX1 (0.00106773), NFIB (0.00153164), NFIC (0.00449107)
limb	ANTTTCCA|TGGAAANT	NFAC2 (3.23806e-05), NFAC1 (0.000235129), NFAC1 (0.000243238), NFAC2 (0.000477478), NFAC3 (0.000527725)
limb	GCCAANNC|GNNTTGGC	NFIA (0.000453438), CXXC1 (0.00760833), IRF5 (0.00786295), HIC1 (0.00845179), NFIC (0.00845179)
limb	CNTGGNCC|GGNCCANG	ZFX (0.00331577), ZFX (0.00952053), PPARD (0.00976171), RXRA (0.0106704), USF1 (0.0141669)
limb	ACNTNTGG|CCANANGT	TWST1 (0.000253346), COT2 (0.000588539), ZBT18 (0.000838423), NR2C1 (0.00125737), TWST1 (0.00137171)
limb	TGGAAAA|TTTTCCA	NFAC3 (5.80182e-06), NFAC2 (1.38469e-05), NFAC1 (2.85729e-05), NFAC1 (6.96052e-05), NFAT5 (0.000190039)
limb	ACATCTG|CAGATGT	TWST1 (3.20882e-05), ZBT18 (0.000200684), TWST1 (0.000211956), NDF1 (0.00109699), TGIF1 (0.00198239)
limb	TGGAAANA|TNTTTCCA	NFAC1 (0.00015476), NFAC3 (0.000156814), NFAC2 (0.000287435), NFAC2 (0.000477478), NFAC1 (0.000554101)
limb	TTCCANA|TNTGGAA	HSF1 (9.46608e-05), STAT6 (0.00333053), NFAC3 (0.00485562), NFAC2 (0.00568304), HSF1 (0.00665018)
limb	CATNTGG|CCANATG	TWST1 (0.000117342), TWST1 (0.000696548), ZBT18 (0.000755208), MSGN1 (0.00233582), MXI1 (0.00234902)
lung	TAAACA|TGTTTA	FOXP2 (2.75026e-05), FOXO3 (3.43781e-05), FOXL2 (4.12536e-05), FOXQ1 (4.81291e-05), FOXJ3 (5.50044e-05)
lung	GTAAAC|GTTTAC	FOXO3 (3.57565e-05), FOXP2 (5.83714e-05), FOXF2 (0.000189313), FOXD1 (0.000226449), FOXA1 (0.000306039)
lung	TGTTTANA|TNTAAACA	FOXL2 (1.77946e-05), FOXO3 (6.10526e-05), FOXK1 (7.16269e-05), FOXP2 (0.000125239), FOXA1 (0.000187853)
lung	CAAACA|TGTTTG	FOXM1 (0.000502455), FOXJ2 (0.00187492), FOXF2 (0.00509825), FOXF1 (0.00586164), GRHL2 (0.00622712)
lung	ANATTCC|GGAATNT	TEAD2 (0.000305099), TEAD1 (0.000358922), NFKB1 (0.000864851), TF65 (0.00116531), TEAD4 (0.00143175)
lung	ACTTCNNG|CNNGAAGT	ELK1 (2.97522e-05), ETV4 (0.000137256), ETS1 (0.000229175), GABPA (0.000296275), ELF2 (0.000421368)
lung	AAGTNCT|AGNACTT	NKX31 (0.000954729), HSF1 (0.00168524), NKX32 (0.00324861), NR1I2 (0.00931488), HNF4A (0.0116877)
lung	AAACTT|AAGTTT	NFAC4 (0.0057021), NFAC2 (0.0135121), NR1I2 (0.0136129), NR1I3 (0.01552), FOXF1 (0.0157289)
lung	ANNTAAAC|GTTTANNT	FOXL2 (2.75026e-05), FOXF2 (8.74114e-05), FOXC1 (0.000112517), FOXD3 (0.000174815), FOXQ1 (0.000196335)
lung	AGNAAACA|TGTTTNCT	FOXM1 (3.97411e-05), FOXP2 (5.30237e-05), FOXC1 (6.3585e-05), FOXF2 (0.000106045), FOXD3 (0.000168925)
forebrain	CAGATGG|CCATCTG	NDF1 (8.33035e-06), NDF2 (1.36443e-05), NGN2 (2.04664e-05), ATOH1 (5.22072e-05), OLIG2 (0.00011425)
forebrain	CAATTA|TAATTG	HXD4 (6.55593e-05), MSX3 (8.25055e-05), MSX2 (0.000159922), NOBOX (0.000277279), PAX3 (0.000314118)
forebrain	ATTNGCA|TGCNAAT	PO2F2 (0.000113231), PO5F1 (0.000152561), PO5F1 (0.000226449), NFIA (0.00107691), NANOG (0.00117295)
forebrain	CNAATTA|TAATTNG	VSX2 (4.12536e-05), NOBOX (0.000239873), LHX2 (0.000411167), MSX3 (0.000539901), MSX2 (0.000591872)
forebrain	TAATTA|TAATTA	VSX2 (4.62738e-05), DLX5 (0.000175831), DLX2 (0.000200822), ZFHX3 (0.000342981), DLX3 (0.00043981)
forebrain	AATTAG|CTAATT	VSX2 (4.81291e-05), LHX2 (0.00090283), PAX3 (0.000919718), PRRX2 (0.000932777), LHX3 (0.00115614)
forebrain	ATGNNAAT|ATTNNCAT	PO2F2 (8.74114e-05), PO5F1 (9.81855e-05), PO5F1 (0.000126581), NANOG (0.000799352), TEF (0.0015726)
forebrain	ACANATNG|CNATNTGT	NDF1 (0.000215369), MSGN1 (0.000596751), NDF2 (0.000699351), OLIG2 (0.000899855), NGN2 (0.00132875)
forebrain	ATGCNAA|TTNGCAT	PO2F2 (0.000113231), PO5F1 (0.000145922), PO5F1 (0.000152561), SOX2 (0.000226449), NANOG (0.00131238)
forebrain	ATGAAT|ATTCAT	PIT1 (0.000392684), PO3F2 (0.000588968), HBP1 (0.00177476), PO3F1 (0.00214113), PO3F2 (0.00336555)
midbrain	TATTCA|TGAATA	PIT1 (0.000243255), PO3F2 (0.000588968), PO3F1 (0.00214113), PO3F2 (0.00281623), PO5F1 (0.00612912)
midbrain	AATAAT|ATTATT	HNF1A (0.000771274), ZFHX3 (0.00104424), SOX13 (0.00284408), NKX61 (0.00300147), HNF1B (0.0040557)
midbrain	ATGNNAAT|ATTNNCAT	PO2F2 (8.74114e-05), PO5F1 (9.81855e-05), PO5F1 (0.000126581), NANOG (0.000799352), TEF (0.0015726)
midbrain	GATNAAA|TTTNATC	PITX2 (0.00144474), DDIT3 (0.00160422), TF7L2 (0.0023944), TF7L1 (0.00269184), TCF7 (0.00272135)
midbrain	ATTTNNAT|ATNNAAAT	PO2F2 (8.43478e-05), PO5F1 (0.000175947), PO5F1 (0.000426445), TEF (0.00121817), NANOG (0.00122567)
midbrain	ATTTNAA|TTNAAAT	PO5F1 (0.0032962), NKX61 (0.0070367), MSX3 (0.00785674), PO2F2 (0.00897796), NANOG (0.0103888)
midbrain	ATCAAA|TTTGAT	TF7L2 (0.000174815), TF7L1 (0.000616687), PBX1 (0.0010276), ARI3A (0.0011779), TCF7 (0.00126439)
midbrain	ATTATNC|GNATAAT	SOX13 (0.00282994), PO6F1 (0.00342192), DLX2 (0.00360463), DBP (0.0066691), CEBPD (0.00852844)
midbrain	ATTATNNA|TNNATAAT	HNF1A (0.000617067), ZFHX3 (0.000696281), NFIL3 (0.00118055), DBP (0.00179316), BARX2 (0.00187131)
midbrain	AATNNAAT|ATTNNATT	PROP1 (2.64424e-05), ALX1 (0.000105434), SMCA5 (0.000302552), PBX1 (0.000792277), FOXP3 (0.00108581)
hindbrain	TAATNAA|TTNATTA	HXC6 (5.94945e-05), HXB8 (0.000285826), HXD13 (0.000285826), HXA5 (0.000308581), PRRX2 (0.000366691)
hindbrain	ATNNATCA|TGATNNAT	HXB7 (2.0627e-05), HXA9 (2.75026e-05), HXB8 (2.75026e-05), HXB6 (4.12536e-05), HXC6 (5.50044e-05)
hindbrain	CNNTGGCA|TGCCANNG	RFX3 (6.9625e-05), RFX2 (0.000135895), RFX1 (0.000262148), RFX1 (0.000364763), RFX2 (0.000364763)
hindbrain	CGNNNAGC|GCTNNNCG	
hindbrain	AGCNNNCG|CGNNNGCT	HINFP (0.00467398),
hindbrain	AATTGNNT|ANNCAATT	PAX3 (0.000131114), BARX1 (0.000399756), MSX3 (0.000591434), PROP1 (0.000722329), ALX1 (0.00104774)
hindbrain	CNCGCA|TGCGNG	AHR (0.00137131), GCM1 (0.00412901), PAX2 (0.00674429), KAISO (0.00926575), EGR1 (0.0105984)
hindbrain	CTAATNA|TNATTAG	VSX2 (4.12536e-05), HXD13 (0.000399756), LHX2 (0.000722329), LHX3 (0.00108375), PRRX2 (0.00120248)
hindbrain	CANCGC|GCGNTG	REST (0.00987728),
hindbrain	ATNCATC|GATGNAT	HXA1 (9.05858e-05), HXB1 (0.000331462), DDIT3 (0.00703321), HXB7 (0.00703321), HXA9 (0.00790298)

Supplementary Table 26: Code word matches with motifs from the HOCOMOCO data base of mouse transcription factors [22]. Tomtom [23] was used to search for matching motifs. Corresponding p-values are shown in brackets.

	ATAC	H3K27ac	H3K4me1	H3K4me3	H3K9me3	H3K27me3	RNA	Control
Forebrain	ENCFF695FLH<br>ENCFF572CMB	ENCFF761HMI<br>ENCFF236VKK	ENCFF924YFS<br>ENCFF551NRH	ENCFF911RYA<br>ENCFF755BPV	ENCFF204RVT<br>ENCFF403OKB	ENCFF460TKS<br>ENCFF926ANX	ENCFF798GDC<br>ENCFF598ESF	ENCFF901RYX<br>ENCFF880SQD
Hindbrain		ENCFF413NTF<br>ENCFF156DKT	ENCFF989KUF<br>ENCFF891VNO	ENCFF884HYZ<br>ENCFF955BLP	ENCFF170JCI<br>ENCFF485DDP	ENCFF642MWW<br>ENCFF187EVU	ENCFF630JFR<br>ENCFF672XLI	ENCFF123XKF<br>ENCFF801CNP
Midbrain	ENCFF060YWL<br>ENCFF100IBX	ENCFF054UNT<br>ENCFF892TDG	ENCFF526EGV<br>ENCFF190ZOW	ENCFF960GUP<br>ENCFF196TGU	ENCFF986SRM<br>ENCFF958RLB	ENCFF318OLQ<br>ENCFF626OTI	ENCFF785FFD<br>ENCFF056VIQ	ENCFF643WUI<br>ENCFF916TFR
Heart		ENCFF576RBC<br>ENCFF261KJB	ENCFF839EWF<br>ENCFF156UJE	ENCFF978DEK<br>ENCFF292UUU	ENCFF502UOP<br>ENCFF262FYW	ENCFF275AKZ<br>ENCFF420BPA	ENCFF951VYO<br>ENCFF143ATC	ENCFF487AIV<br>ENCFF642FOT
Kidney	ENCFF036RNM<br>ENCFF477SZP	ENCFF650CDV<br>ENCFF831JKI	ENCFF071PID<br>ENCFF167AOI	ENCFF262SUZ<br>ENCFF407ALD	ENCFF148BAU<br>ENCFF110BCP	ENCFF139UWI<br>ENCFF977IBX	ENCFF258QXM<br>ENCFF522LYF	ENCFF627RIT<br>ENCFF285XPR
Limb		ENCFF156ZFI<br>ENCFF052AZF	ENCFF173RDF<br>ENCFF295BOL	ENCFF148HFT<br>ENCFF536ZMQ	ENCFF894UZN<br>ENCFF011YLZ	ENCFF003PZM<br>ENCFF518UCO	ENCFF445SGU<br>ENCFF223JGE	ENCFF031OYA<br>ENCFF514GVM
Liver		ENCFF027UTM<br>ENCFF826GUO	ENCFF783RDN<br>ENCFF141HFI	ENCFF031RFH<br>ENCFF492LOP	ENCFF477WQY<br>ENCFF858QZZ	ENCFF869LGO<br>ENCFF031MFD	ENCFF789CGL<br>ENCFF043NJH	ENCFF209HPI<br>ENCFF634NAV
Lung		ENCFF561MSI<br>ENCFF497ZYH	ENCFF338GKD<br>ENCFF555LDI	ENCFF401RKM<br>ENCFF669NCH	ENCFF380FFY<br>ENCFF222SXN	ENCFF788XZB<br>ENCFF801BAX	ENCFF870FAN<br>ENCFF699ZJN	ENCFF079JAD<br>ENCFF269JKO

Supplementary Table 27: ENCODE bam accession codes

Hindbrain	ENCFF761JHA	ENCFF181OVJ	ENCFF213OUA	ENCFF709CLT
Heart	ENCFF829XFO	ENCFF694SPD	ENCFF385WYM	ENCFF051GLX
Limb	ENCFF629FRA	ENCFF222KJL	ENCFF488EFS	ENCFF917DTZ
Liver	ENCFF329VCX	ENCFF290ZBP	ENCFF341HRL	ENCFF489XAT
Lung	ENCFF507MHY	ENCFF217WJG	ENCFF159YPF	ENCFF940GZL

Supplementary Table 28: ENCODE ATAC fastq accession codes

References

[1] Khan, A., Fornes, O., Stigliani, A., Gheorghe, M., Castro-Mondragon, J. A., van der Lee, R., Bessy, A., Cheneby, J., Kulkarni, S. R., Tan, G., et al. (2017) JASPAR 2018: update of the open-access database of transcription factor binding profiles and its web framework. *Nucleic acids research,* **46**(D1), D260–D266.

[2] Weirauch, M. T., Cote, A., Norel, R., Annala, M., Zhao, Y., Riley, T. R., Saez-Rodriguez, J., Cokelaer, T., Vedenko, A., Talukder, S., et al. (2013) Evaluation of methods for modeling transcription factor sequence specificity. *Nature biotechnology,* **31**(2), 126.

[3] Barski, A., Cuddapah, S., Cui, K., Roh, T.-Y., Schones, D. E., Wang, Z., Wei, G., Chepelev, I., and Zhao, K. (2007) High-resolution profiling of histone methylations in the human genome. *Cell,* **129**(4), 823–837.

[4] Heintzman, N. D., Stuart, R. K., Hon, G., Fu, Y., Ching, C. W., Hawkins, R. D., Barrera, L. O., Van Calcar, S., Qu, C., Ching, K. A., et al. (2007) Distinct and predictive chromatin signatures of transcriptional promoters and enhancers in the human genome. *Nature genetics,* **39**(3), 311.

[5] Heintzman, N. D., Hon, G. C., Hawkins, R. D., Kheradpour, P., Stark, A., Harp, L. F., Ye, Z., Lee, L. K., Stuart, R. K., Ching, C. W., et al. (2009) Histone modifications at human enhancers reflect global cell-type-specific gene expression. *Nature,* **459**(7243), 108.

[6] Heinz, S., Romanoski, C. E., Benner, C., and Glass, C. K. (2015) The selection and function of cell type-specific enhancers. *Nature reviews Molecular cell biology,* **16**(3), 144.

[7] Calo, E. and Wysocka, J. (2013) Modification of enhancer chromatin: what, how, and why?. *Molecular cell,* **49**(5), 825–837.

[8] Zhu, J., He, F., Hu, S., and Yu, J. (2008) On the nature of human housekeeping genes. *Trends in genetics,* **24**(10), 481–484.

[9] Bird, A. (2002) DNA methylation patterns and epigenetic memory. *Genes & development,* **16**(1), 6–21.

[10] Voss, S. D., Schlokat, U., and Gruss, P. (1986) The role of enhancers in the regulation of cell-type-specific transcriptional control. *Trends in Biochemical Sciences,* **11**(7), 287–289.

[11] Deaton, A. M. and Bird, A. (2011) CpG islands and the regulation of transcription. *Genes & development,* **25**(10), 1010–1022.

[12] Roider, H. G., Lenhard, B., Kanhere, A., Haas, S. A., and Vingron, M. (2009) CpG-depleted promoters harbor tissue-specific transcription factor binding signals-implications for motif overrepresentation analyses. *Nucleic acids research,* **37**(19), 6305–6315.

[13] Johnson, P. F. and McKnight, S. L. (1989) Eukaryotic transcriptional regulatory proteins. *Annual review of biochemistry,* **58**(1), 799–839.

[14] Mitchell, P. J. and Tjian, R. (1989) Transcriptional regulation in mammalian cells by sequence-specific DNA binding proteins. *Science,* **245**(4916), 371–378.

[15] Wasylyk, B., Wasylyk, C., Flores, P., Begue, A., Leprince, D., and Stehelin, D. (1990) The c-ets proto-oncogenes encode transcription factors that cooperate with c-Fos and c-Jun for transcriptional activation. *Nature,* **346**(6280), 191.

[16] Janson, L. and Pettersson, U. (1990) Cooperative interactions between transcription factors Sp1 and OTF-1.. *Proceedings of the National Academy of Sciences,* **87**(12), 4732–4736.

[17] Kel, O. V., Romaschenko, A. G., Kel, A. E., Wingender, E., and Kolchanov, N. A. (1995) A compilation of composite regulatory elements affecting gene transcription in vertebrates. *Nucleic acids research,* **23**(20), 4097–4103.

[18] Pilpel, Y., Sudarsanam, P., and Church, G. M. (2001) Identifying regulatory networks by combinatorial analysis of promoter elements. *Nature genetics,* **29**(2), 153.

[19] Zhu, Z., Shendure, J., and Church, G. M. (2005) Discovering functional transcription-factor combinations in the human cell cycle. *Genome research,* **15**(6), 848–855.

[20] van Bömmel, A., Love, M. I., Chung, H.-R., and Vingron, M. (2018) coTRaCTE predicts co-occurring transcription factors within cell-type specific enhancers. *PLoS computational biology,* **14**(8), e1006372.

[21] Bien, J., Taylor, J., and Tibshirani, R. (2013) A lasso for hierarchical interactions. *Annals of statistics,* **41**(3), 1111.

[22] Kulakovskiy, I. V., Vorontsov, I. E., Yevshin, I. S., Sharipov, R. N., Fedorova, A. D., Rumynskiy, E. I., Medvedeva, Y. A., Magana-Mora, A., Bajic, V. B., Papatsenko, D. A., et al. (2018) HOCOMOCO: towards a complete collection of transcription factor binding models for human and mouse via large-scale ChIP-Seq analysis. *Nucleic acids research,* **46**(D1), D252–D259.

[23] Bailey, T. L., Boden, M., Buske, F. A., Frith, M., Grant, C. E., Clementi, L., Ren, J., Li, W. W., and Noble, W. S. (2009) MEME SUITE: tools for motif discovery and searching. *Nucleic acids research,* **37**(suppl_2), W202–W208.
